# Ultrafast Photoexcitation Induced Nonthermal Lattice Expansion in 2D Perovskite

**DOI:** 10.1002/advs.202510954

**Published:** 2025-10-21

**Authors:** Xiangyu Chen, Jiakang Zhou, Yunfan Yue, Zhongle Zeng, Xuewen Wang

**Affiliations:** ^1^ Center of Femtosecond Laser Manufacturing for Advanced Materials and Devices State Key Laboratory of Advanced Technology for Materials Synthesis and Processing Wuhan University of Technology Wuhan 430070 P. R. China; ^2^ Sanya Science and Education Innovation Park of Wuhan University of Technology Sanya 572025 P. R. China; ^3^ National Energy Key Laboratory for New Hydrogen‐Ammonia Energy Technologies Foshan Xianhu Laboratory Foshan 528000 P. R. China

**Keywords:** 2D perovskite, lattice expansion, passivation, ultrafast photoexcitation

## Abstract

Light‐induced lattice change of perovskites is found to be critical for device performance. However, the underlying mechanism is still not fully understood. Here, real‐time time‐dependent density functional theory (rt‐TDDFT) calculations along with experiments are utilized to discern the photo‐induced electronic and structural changes in 2D Dion‐Jacobson (DJ) perovskites, showing that photoexcitation drives the non‐thermal lattice expansion, leading to the increase in bandgap. The ultrafast photoexcitation effect is found to improve the film crystallinity and charge transport, which brings a longer carrier lifetime and slower carrier recombination rate, leading to remarkable improvement of photovoltaic performance. These results not only provide insights into the photo‐physics of perovskites, but also show the potential of a new passivation technique for perovskite optoelectronics via ultrafast photoexcitation.

## Graphical abstract

Photo‐induced lattice expansion in 2D Dion‐Jacobson (DJ) perovskites is a non‐thermal process, resulting in the more relaxed local lattice strain and the larger bandgap. It activates a self‐passivation mechanism, effectively diminishing trap state density. The synergy of these effects leads to suppressed non‐radiative recombination and prolonged charge carrier lifetimes, culminating in enhanced photovoltaic efficiency and stability.

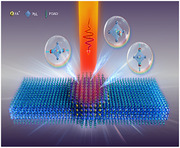

## 1 Introduction

Metal halide perovskite solar cells (PSCs) have long been a promising candidate in the photovoltaic industry owing to their excellent optoelectronic properties^[^ ^1^ ^]^ exceeding some silicon solar cells^[^ ^2^ ^]^ and their low‐cost fabrication processes.^[^ ^3^ ^]^ However, the stability, particularly the photostability performance, which can't survive from encapsulation processes, is still the predominant challenge for commercial applications. Under light illumination, perovskites undergo various structural changes, including the induction of metastable defect states,^[^ ^4^ ^]^ ion migration^[^ ^5^ ^]^ and phase segregation,^[^ ^6^ ^]^ or generation of lattice strain distributions,^[^ ^7^ ^]^ and emergence of macroscopic photostriction phenomenon.^[^ ^8^ ^]^ Most of these photo‐effects have detrimental influences on the film morphology, bandgap structures, and further lead to decreased carrier lifetime and mobility. These factors ultimately trigger hysteretic behavior, diminish device stability, and eventually result in device degradation. Notably, few experiments have reported the positive effect on the device optoelectronic properties^[^ ^9^ ^]^ and photostability.^[^ ^10^ ^]^ However, the underlying mechanism is still under debate.^[^ ^9^, ^11^, ^12^ ^]^

Numerous approaches have been attempted to understand the structural dynamics^[^ ^13^ ^]^ within perovskites under photoexcitation.^[^ ^14^, ^15^ ^]^ It is noted that electrons are excited from the valence band to populate the conduction band within femtosecond timescales and cooled in picosecond timescales, which changes the charge distribution affecting the strength of covalent lead─halide bonds^[^ ^8^ ^]^ and hydrogen bonds between organic moiety and iodide.^[^ ^16^ ^]^ With high‐density carriers excited, the Coulomb screening effect then plays a critical role, which significantly screens the carrier‐phonon interaction, further leading to the deformation of lead─halide─lead bond angles or lead─halide bond lengths with octahedron distortion or expansion at ultrafast timescales.^[^ ^17^ ^]^ The phenomenon of photoinduced lattice expansion has been observed in 3D perovskites, including FA_0.7_MA_0.25_Cs_0.05_PbI_3_ perovskites,^[^ ^9^ ^]^ FA_0.79_MA_0.16_Cs_0.05_Pb(I_0.83_Br_0.17_)_3_ perovskites,^[^ ^18^ ^]^ and 2D Ruddlesden–Popper BA_2_MA_2_Pb_3_Br_10_ perovskites.^[^ ^19^ ^]^ The final structural deformation considerably affects the electronic bandgap, carrier recombination rate,^[^ ^20^ ^]^ as well as the defect relaxation process of perovskites,^[^ ^21^ ^]^ which turns to dominate the device performance. Employing external energy injection into perovskite films can also lead to structural change and passivation effect such as oxygen plasma^[^ ^22^ ^]^ and cryogenic e‐beam,^[^ ^23^ ^]^ but these continuous energy injection methods lack the ability to investigate ultrafast timescale structural dynamics. Considering the state‐of‐the‐art of control material properties by ultrashort light pulses,^[^ ^24^ ^]^ these observations provide a clue to tune the properties of perovskites by ultrafast photoexcitation.^[^ ^25^ ^]^

Herein, we utilize real‐time time‐dependent density functional theory (rt‐TDDFT) calculations along with experiments to discern the photoinduced electronic and structural change in 2D Dion‐Jacobson (DJ) perovskites. Under ultrafast photoexcitation, the Pb─I bond length increases, resulting in the lattice expansion, which then leads to the increase of bandgap. We find this structural change also improves the crystal crystallinity and minimizes surface defect densities, playing a role of surface passivation, which brings a longer carrier life and slower carrier recombination rate, leading to an improvement of photovoltaic performance. Consequently, the power conversion efficiency (PCE) of the optimized device is enhanced from 12.43% to 14.05%, which the ratio is increased by 12.13% relative to the unexcited counterpart. Besides, the corresponding unencapsulated devices can sustain more than 95% of their original efficiency after being stored in an atmospheric environment for 1600 h.

## 2 Results and Discussion

**Figure 1:**
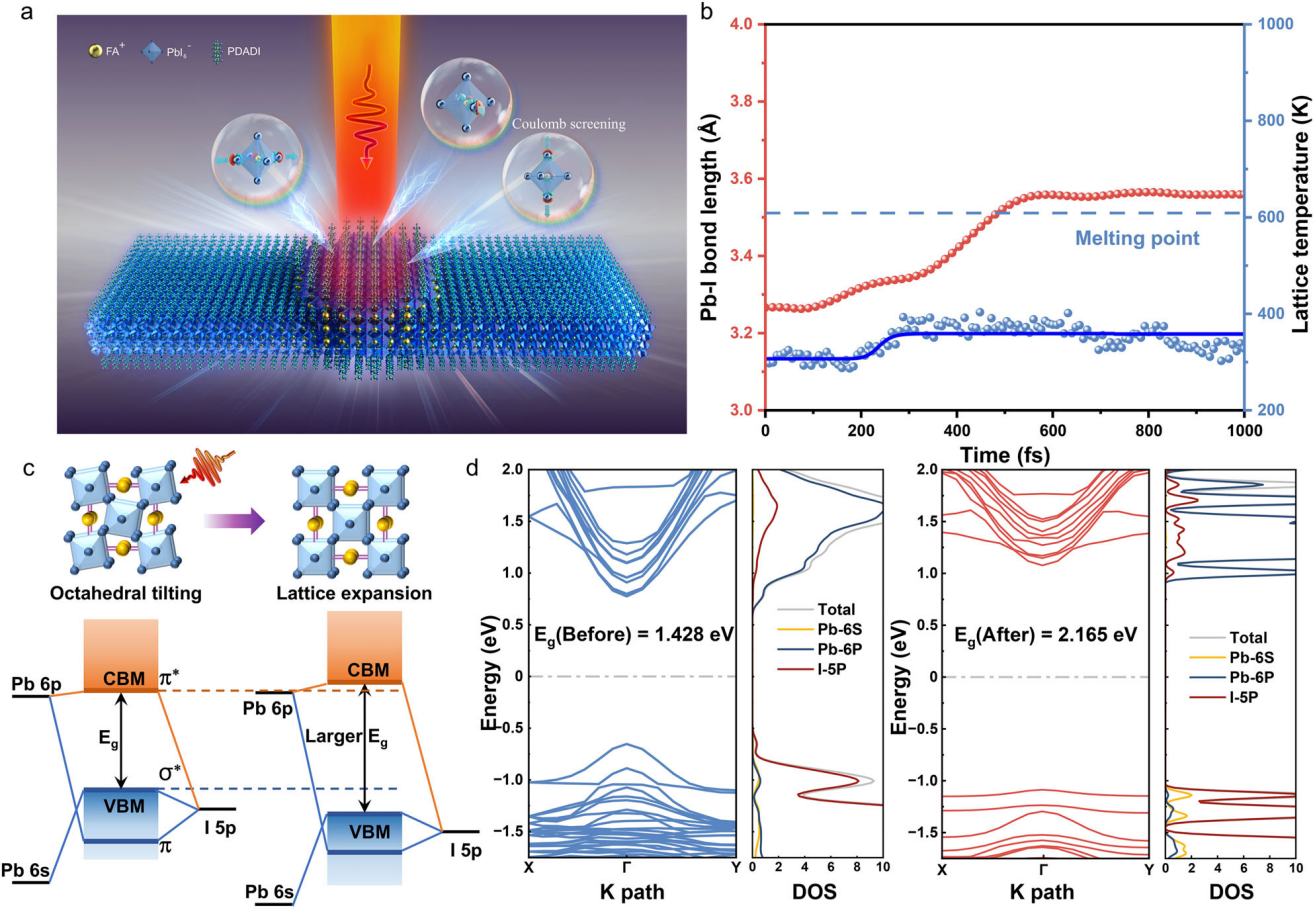
The mechanism of lattice expansion of quasi‐2D (PDA)(FA)_n‐1_Pb_n_I_3n+1_ (n = 4) perovskite induced by ultrafast photoexcitation. a) The schematic of the lattice expansion process triggered by ultrafast photoexcitation. b) Evolution of the Pb─I average bond length (red scatter) and lattice temperature (blue scatter) with time upon 1.266 eV photon energy photoexcitation. The blue line is obtained from the logistic fit of the lattice temperature. c) Pristine and photoexcited perovskite lattice structure, along with the corresponding energy level schematic. d) Band structure and density of states (DOS) of pristine quasi‐2D perovskite (blue line) and photoexcited perovskite (red line) at 120 fs calculated by Perdew–Burke–Ernzerhof (PBE) functional, respectively.

The schematic of perovskite lattice expansion induced by ultrafast photoexcitation is shown in **Figure** **1**a. We use an ultrashort pulse laser beam with a pulse duration of 260 fs, photon energy of 1.204 eV, and photon flux of 1.06  10^17^ photons cm^−2^ below the threshold as displayed in Figures S1 and S2 (Supporting Information) to scan the perovskite surface. The high photon flux enables multiphoton absorption, exciting substantial carriers from the valence band to the conduction band.^[^ ^26^ ^]^ The resultant Coulomb screening effect significantly screens the carrier–phonon and phonon–phonon interaction, leading to the transient prolongation of Pb─I bonds.^[^ ^27^ ^]^ Due to the excessive grain boundaries, the film will preserve part of the lattice expansion after relaxation, accompanied by modulating the optoelectronic properties of the relaxed photoexcited systems.

We utilize rt‐TDDFT methods to explore the structure deformation during photoexcitation. The crystal structures of the (PDA)(FA)_n‐1_Pb_n_I_3n+1_ perovskite are summarized in Table S1 (Supporting Information). Before photoexcitation, the electron density around iodine atoms is higher than that around lead atoms in the octahedral structure shown in Figure S3 (Supporting Information). However, upon excitation, the electrons belonging to iodine atoms are excited and transferred to lead atoms, resulting in the photogenerated electrons being primarily distributed around lead atoms, while the photogenerated holes are mainly distributed around iodine atoms (Figures S4–S6, Supporting Information), generating a Coulomb screening effect which elongates the lead‐halide bond. Such a change of the charge distribution in lead and iodine atoms can still be observed by the shift of the binding energy of the atoms in X‐ray photoelectron spectroscopy (XPS) in Figure S7 (Supporting Information) after the system relaxed. In Figure 1b, the Pb─I bond starts to elongate at 80 fs when the number of photogenerated electron‐hole pairs are no longer increasing (as shown in Figure S4, Supporting Information) after photoexcitation from 3.26 Å and continually reaches ≈3.56 Å, stretching by ≈0.3 Å, finally leading to an expansion in the lattice. Significantly, we found that with higher photon energy far from the bandgap at non‐resonant excitation, the spatial localization distribution of excess photoexcited carriers concentrated at the band edge aggravate dynamic instability of the lattice, which in turn leading to the density of localized excited carriers surpasses the needed density of phonon softening,^[^ ^28^ ^]^ leading to lattice temperature more than melting point and eventually causing the self‐amplified melting of the system as displayed in Figures S8 and S9 (Supporting Information). While with lower photon energy close to the bandgap at near‐resonant excitation, despite the electron density is high, the bandgap remains existing and the lattice temperature is far below the melting point (610 K) (Figure S10, Supporting Information) different from non‐resonant excitation during the whole simulation process, indicating the ultrafast photoexcitation induced lattice expansion is a nonthermal process.

We further investigate the electronic transition process during photoexcitation. From Figure 1c,d, and Figure S11 (Supporting Information), it can be seen that the valence band maximum (VBM) of perovskite is dominated by I 5p and Pb 6s hybrid antibonding orbitals, while the conduction band minimum (CBM) is predominantly by nonbonded Pb 6p orbitals and a little contribution of Pb 6p and I 5p hybrid antibonding orbitals. When the photon energy is much higher than the bandgap, valence electrons from the antibonding and the deeper bonding of I 5p and Pb 6s/6p hybrid orbitals will be excited. When only exciting electrons at the VBM, the Coulomb repelling force between Pb and I atoms will be reduced, leading to a shortening of the Pb─I bond and resulting in lattice contraction. In contrast, the excitation of electrons in the bonding states will generate the opposite effect, causing lattice expansion.^[^ ^29^ ^]^ When the perovskite system is excited with high photon energy and more electrons in the bonding states are excited than that in the VBM, the overall effect will be dominated by lattice expansion. Accompanied by lattice expansion through Pb─I bond stretching, the overlap between the antibonding orbitals of I 5p and Pb 6s will be reduced, resulting in the energy level of VBM moving downwards and CBM moving with a subtle upshift.^[^ ^30^ ^]^ Consequently, the bandgap widens from 1.428 to 2.165 eV at 120 fs after photoexcitation, as shown in Figure 1d; Figure S12 (Supporting Information). This lattice expansion effectively mitigates lattice distortions, enhances structural symmetry, and drives the relaxation of localized strain.

**Figure 2:**
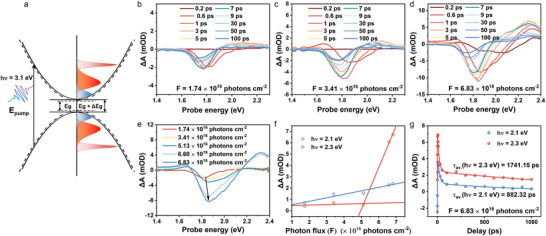
Carrier dynamics of (PDA)(FA)_n‐1_Pb_n_I_3n+1_ (*n* = 4) perovskite film upon photoexcitation at various delay time. a) A schematic diagram of a carrier undergoing thermalization and gradually cooling to the modified band edge after the pump pulse excitation at 3.1 eV. Carrier dynamics of perovskite film at fast delay time varying from 0.2 ps to 100 ps of photon flux F = 1.74 × 10^16^ photons cm^−2^ (8.65 mJ cm^−2^) b) and F = 3.41 × 10^16^ photons cm^−2^ (16.93 mJ cm^−2^) c) and F = 6.83 × 10^16^ photons cm^−2^ (33.87 mJ cm^−2^) d). A new feature occurs at 2.3 eV when the carrier density is extremely high shown in (d). e) The initial position of the GSB signature scaling with carrier density with delay time at 0.6 ps, accompanied by blueshift. f) Graph of ΔA as a function of photon flux at hν = 2.1 and 2.3 eV with a delay time at 3 ps. Notably, the PIA at 2.3 eV can be observed only when the photon flux reach to the threshold F_th_ = 5.12 × 10^16^ photons cm^−2^. g) Graph of ΔA as a function of delay time at hν = 2.1 and 2.3 eV of F = 6.83 × 10^16^ photons cm^−2^, where the τ_av_ at 2.1 and 2.3 eV is 882.32 and 1741.15 ps, respectively.

To investigate the deep excitation process of bonded electrons in the VB, we utilize a 400 nm (3.1 eV) pump pulse to study the dynamics of excited carriers under lattice expansion after photoexcitation. The excited carriers rapidly undergo thermalization within a few hundred femtoseconds, obeying Maxwell–Boltzmann distribution, and gradually cool down to the modified band edge.^[^ ^31^ ^]^ The schematic process is shown in **Figure** **2**a. From Figure 2b–d and Figures S13 and S14 (Supporting Information), when the photon flux is relatively low, the transient absorption (TA) spectrum exhibits three features including two positive photo‐induced absorption (PIA) features and a negative ground state bleaching (GSB) feature. As the photon flux increases, a new PIA signal occurs at roughly 2.3 eV. Note that the carrier densities corresponding to photon flux are shown in Table S2 (Supporting Information).

The PIA, below the bandgap as a result of ultrafast bandgap renormalization (BGR) generated by photoexcited free carriers, scales with the photon flux and gradually reduces in comparison with the enhancement of the GSB signal. The initial GSB energy is larger than bandgap due to the Burstein–Moss effect,^[^ ^32^, ^33^ ^]^ while the energy and the magnitude of GSB increase with the photon flux starkly (Figure 2e). Besides, the GSB gradually redshifts with the increase of delay time, indicating the lattice obtains energy from hot carriers through carrier–phonon scatting. The PIA features at 2.1 and 2.3 eV above the bandgap are probably caused by the strong absorption from the interaction between photon‐excited carriers and deformed lattices.^[^ ^34^ ^]^ The magnitude of PIA at 2.1 and 2.3 eV is proportional to photon flux in Figure 2f. However, the PIA located at 2.3 eV can be clearly observed only when the photon flux reach to the threshold F_th_ = 5.12 × 10^16^ photons cm^−2^, which may result from the strong structural deformation caused by lattice expansion due to ultrafast intensive photoexcitation, and has the same tendency as the GSB signature before 200 ps, shown in Figure 2b–d and Figures S13 and S14 (Supporting Information). The hot carrier temperature was extracted by fitting the high‐energy tail from 1.85 to 2.05 eV of the GSB signal with the Maxwell–Boltzmann distribution function as shown in Figure S14 (Supporting Information). At higher photon flux, the slope of the cooling curve becomes less steep, indicating a reduction in the carrier cooling rate and a more pronounced hot phonon bottleneck phenomenon. The PIA signals located at 2.1 and 2.3 eV are the sign of ultrafast photoexcitation induced lattice change, showing a long‐time recovering process compared to other processes, with lifetime of 882.32 and 1741.15 ps respectively, as shown in Figure 2g and Table S3 (Supporting Information). In comparison to the lifetime of the 1.65 eV PIA signal that is associated with BGR shown in Figure S15 (Supporting Information), the high‐energy peak exhibits a substantially longer lifetime.

**Figure 3:**
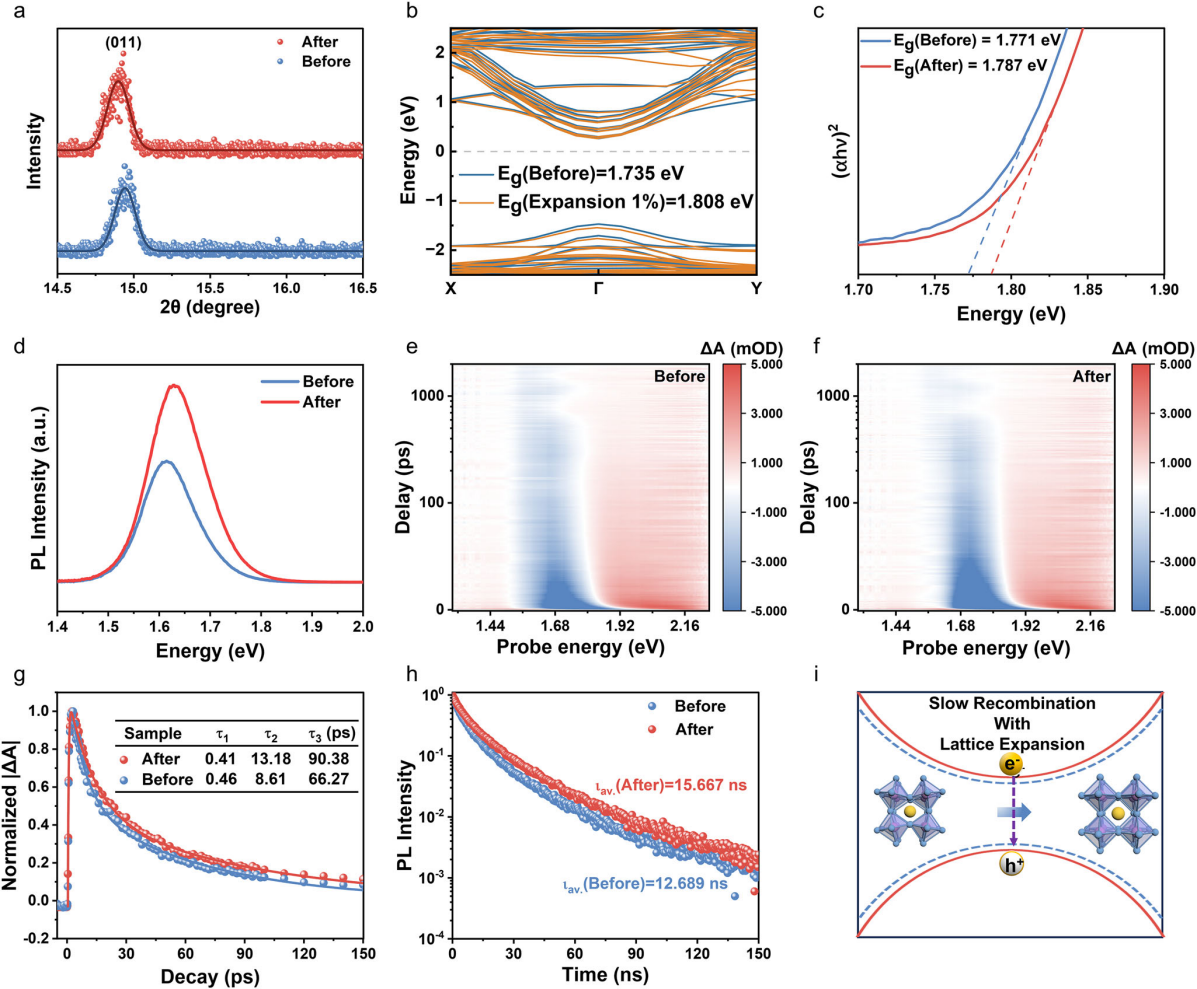
Effect of lattice expansion induced by ultrafast photoexcitation on materials characterization of (PDA)(FA)_n‐1_Pb_n_I_3n+1_ (*n* = 4) perovskite film. a) X‐Ray Diffraction (XRD) patterns of perovskite films before and after the photoexcitation with the photon flux of 1.06 × 10^17^ photons cm^−2^. b) Band structure of perovskite films calculated by Heyd–Scuseria–Ernzerhof (HSE06) functional, where the blue line representing the pristine sample and the orange line representing the sample with 1% volume expansion. c) The Tauc plots of perovskite films before and after the photoexcitation. d) Photoluminescence (PL) spectra of the perovskite film at different conditions. TA spectra of perovskite films without e) and with the photoexcitation f). g)The kinetics analysis of photo‐induced bleaching (PIB) signals with a triple exponential fit of the corresponding perovskite film in (e) and (f). h) Time‐resolved photoluminescence (TRPL) of the perovskite films before and after the photoexcitation. i) A schematic diagram of a slower recombination rate with lattice expansion under photoexcitation.

To further verify the final modification of the lattice structure of 2D DJ perovskite polycrystalline films after the ultrafast photoexcitation, we further utilize XRD to analyze the crystallographic planes spacing. The two domain diffraction peaks at 14.9° and 28.6° correspond to (011) and (022) planes of the 2D DJ phase in Figure S16 (Supporting Information),^[^ ^35^ ^]^ respectively. Both peaks exhibit slight shifts toward lower diffraction angles, validating the octahedron expansion. In detail, the (011) phase moves from 14.942° to 14.898° shown in **Figure** **3**a, corresponding to the increment of 0.017 ± 0.001 Å in interplanar spacing, leading to a 0.8% expansion in volume after photoexcitation. Meanwhile, the elevated Bragg peak intensity demonstrates that the crystallinity of the 2D DJ phase is increased upon photoexcitation. By comparing the static band structure of pristine film with that of the 1% volume expanded film via theoretical calculation by using Heyd–Scuseria–Ernzerhof (HSE06) hybrid functional, we find that the bandgap increases from 1.735 to 1.808 eV after lattice expansion (Figure 3b; Figure S17, Supporting Information), which stems from the downshift of VBM. That is consistent with the experiment observation from the Tauc plots in Figure 3c, showing the bandgap increasing from 1.771 to 1.787 eV after ultrafast photoexcitation.

As shown in photoluminescence (PL) spectra (Figure 3d), the PL intensity is significantly boosted indicating the defect‐mediated non‐radiative recombination is effectively suppressed. Moreover, the peak position of the perovskite film upon photoexcitation is also slightly blueshifted, proving the elevated optical bandgap and indicating a lower trapping density in the perovskite films,^[^ ^36^, ^37^ ^]^ which further validates that ultrafast photoexcitation does have a beneficial effect on defect passivation. The broadening of PL profile implies the occurrence of strong exciton‐phonon interaction, which promotes polaron formation, resulting in a high defect tolerance and long carrier diffusion length.^[^ ^38^ ^]^ Compared to the TA spectra of the pristine and photoexcited perovskite films in Figure 3e with Figure 3f, the position of the GSB feature shows blueshift from 1.712 to 1.731 eV due to the lattice expansion, and the enhancement of the GSB signal lifetime displays a higher excited carrier concentration after ultrafast photoexcitation. We further use a triple‐exponential equation (Table S4, Supporting Information) to fit GSB signal to understand carrier dynamics (Figure 3g), where τ_1_ represents the photocarrier excitation process, τ_2_ is related to the carrier diffusion process, and τ_3_ characterizes the carrier recombination process. The diffusion and recombination kinetic profiles of quasi‐2D (*n* = 4) films after ultrafast photoexcitation are longer than those of untreated films,^[^ ^39^ ^]^ suggesting slower carrier diffusion and a lower carrier recombination rate in the treated film resulting from the interplay between the expanded lattice and mitigated defect centers, which will benefit the photovoltaic performance. Significantly, the average hot carrier lifetimes obtained from GSB signals also increase by ≈20 ps due to the hot‐phonon bottleneck occurring when the carrier density is higher than 10^18^ cm^−3^, slowing down the carrier relaxation process.

Time‐resolved photoluminescence (TRPL) is used to characterize the carrier lifetime in Figure 3h. The average carrier lifetime of (PDA)(FA)_n‐1_Pb_n_I_3n+1_ (*n* = 4) perovskite increases from 12.689 to 15.667 ns, which aligns with Fermi's golden rule. We use the biexponential decay equation to fit TRPL curves as shown in Table S5 (Supporting Information). The fast decay component (τ_1_) associated with the surface trap‐mediated nonradiative recombination is increased from 3.862 to 4.661 ns, while the slow decay component (τ_2_) related to radiative recombination of free carriers is also enhanced from 16.670 to 19.249 ns.^[^ ^40^ ^]^ It shows that the synergistic interplay between bandgap enlargement arising from lattice expansion and effective passivation stemming from photoinduced structure dynamics can boost carrier lifetimes, thus slowing recombination both at the surface and in the bulk and ultimately leading to high‐performance devices (Figure 3i).^[^ ^30^ ^]^

We also demonstratethat ultrafast photoexcitation is beneficial to surface passivation through lattice expansion to optimize the surface morphology via atomic force microscopy (AFM) and scanning electron microscopy (SEM). As depicted in Figure S18 (Supporting Information), the roughness of films decreases from Ra = 11.4 to Ra = 8.77 nm while the average grain size increases from ≈420 to ≈550 nm because of Ostwald ripening after the photoexcitation, indicating the surface becomes flatter and smoother.^[^ ^41^ ^]^ Generally, the film before the ultrafast photoexcitation contains large particles close to micrometers in size and many scattered tiny massive grains, whereas the treated film exhibits a more uniform morphological distribution and larger crystal grains. The larger the grain size is, the less the grain boundary will be, resulting in fewer surface defects, thus increasing the carrier lifetime. It should be noted that after the photoexcitation, the contact angle gradually becomes larger (Figure S19, Supporting Information), indicating the film surface becomes more hydrophobic, which does good to fabricating hole transport layer (HTL) on top of perovskites with spin‐coating methods and indirectly enhancing the stability of devices. These results demonstrate that ultrafast photoexcitation does modify interfaces, which can not only effectively enlarge grain sizes, but also improve overall film qualities, which is beneficial to fabricating excellent photovoltaic devices and elevating the PCE and stability.

**Figure 4:**
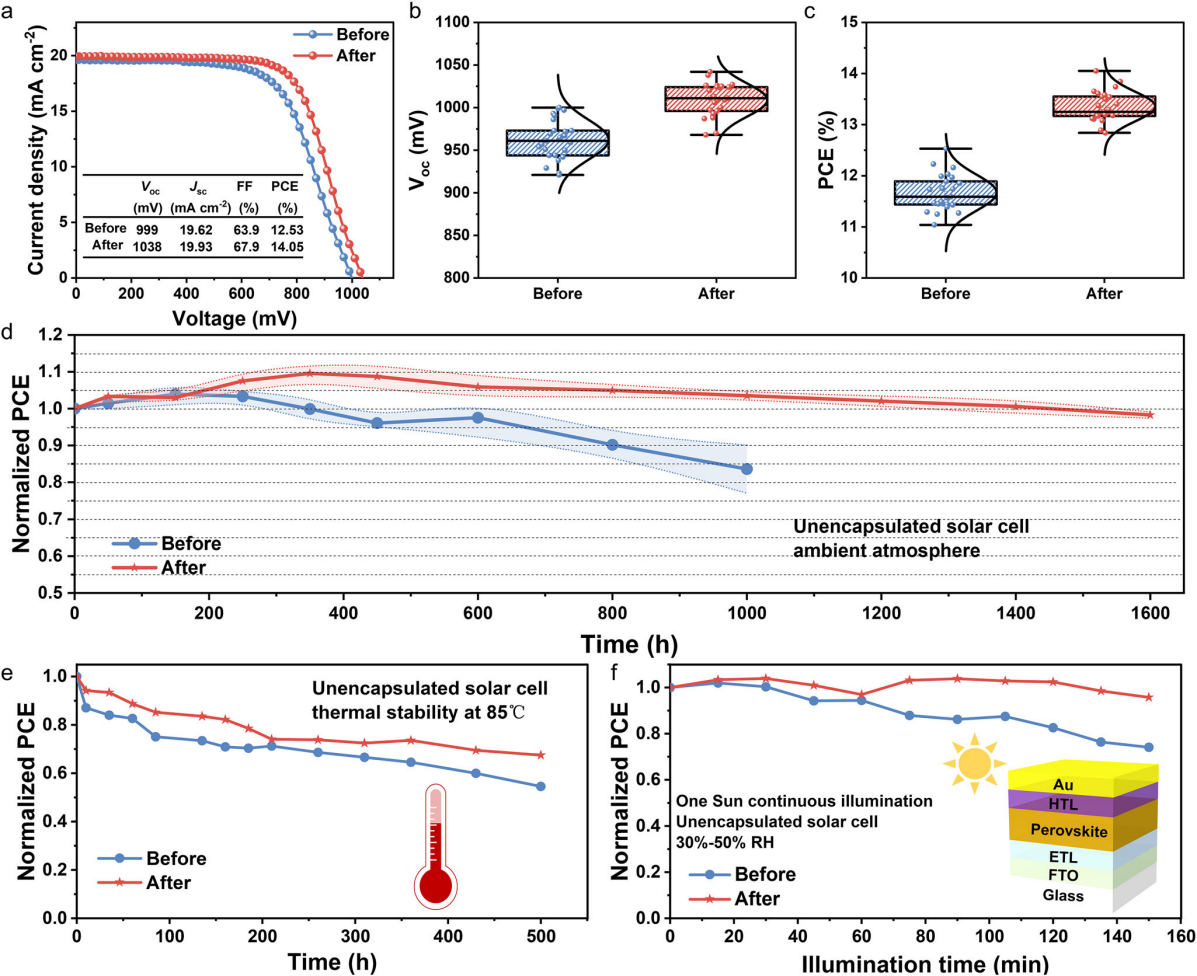
Device performance and stability. a) PCE of (PDA)(FA)_n‐1_Pb_n_I_3n+1_(*n* = 4) PSCs before and after ultrafast photoexcitation. Statistics of *V*_OC_ b) and PCE c) respectively, for 25 devices. d) Normalized PCE of the unencapsulated PSCs under ambient air condition lasting 1500 h. e) Thermal stability of PSCs before and after photoexcitation at 85 °C for 500 h. f) Normalized PCE of the unencapsulated before and after photoexcitation treated PSCs with 150 min illumination time.

To explore the influence of ultrafast photoexcitation‐induced passivation on the photovoltaic performance, we utilize a typical n‐i‐p cell stack of FTO/SnO_2_/(PDA)(FA)_n‐1_Pb_n_I_3n+1_ (*n* = 4)/Spiro‐OMeTAD/Au structure without and with photoexcitation to characterize the devices performance. From **Figure** **4**a, we find the champion PCE of the device without and with photo‐induced lattice expansion is enhanced from 12.53% to 14.05%, representing an improvement of ≈12.13%, mainly owing to the increased *V*_OC_ and fill factor (FF). As confirmed by Figure 4b,c and Figure S20 (Supporting Information), the statistical average *V*_OC_ and FF among 25 devices are enhanced from 962.32 to 1008.92 meV and from 62.08% to 67.11%, respectively, contributing to the increment of average PCE from 11.67% to 13.35%. The external quantum efficiency data shown in Figure S21 (Supporting Information) of the corresponding devices is in good agreement with the *J*_SC_ tested by the current density‐voltage curves.

Moreover, we fabricate 2D perovskite (*n* = 2) devices to further demonstrate the versatility of the lattice expansion in enhancing device efficiency. As shown in Figure S22 and Table S6 (Supporting Information), the results indicate that lattice expansion also leads to increased *V*_OC_ and FF, resulting in an enhancement of PCE from 2.63% to 4.43%. An obvious elevation in *V*_OC_ and FF in both 2D perovskite (*n* = 2) and quasi‐2D perovskite (*n* = 4) can be observed, owing to the synergetic interaction among photoexcitation‐induced surface passivation and enhanced lattice relaxation. The former effect improves surface grain homogeneity and reduces surface defect states, while the latter effect mitigates non‐radiative recombination to some extent. These results demonstrate that lattice expansion effectively alleviates charge recombination at the interface between perovskite and HTL by suppressing non‐radiative capture through enhanced lattice relaxation during the transition of the defect charge states.^[^ ^21^ ^]^ Furthermore, the similar or slightly enhanced *J*_SC_ indicates that the photoexcitation‐induced passivation causes no damage to the film. The narrower distribution of performance parameters for photoexcitation treated devices further confirms the reproducibility of this technique.

Besides, ultrafast photoexcitation‐induced passivation not only enhances the device performance but also improves the device stability, as shown in Figure 4d. The long‐term stability of passivation treated unencapsulated devices maintains more than 98% of their initial PCE over 1600 h versus 83% of before counterpart after 1000 h, measured under ambient air conditions of 20% ± 5% relative humidity (RH). Meanwhile, we compare XRD pattern data of the corresponding perovskite thin film taken before and after being stored 1000 h in ambient air conditions (Figure S23, Supporting Information). After 1000 h, the peak intensity ratio of the photoexcited perovskite (011) to the untreated perovskite (011) Bragg peak is 2.78, which is much higher than 1.66 in the corresponding pristine perovskite film. Similarly, the peak intensity ratio for the (022) diffraction peak increased from 1.8 (pristine) to 3.42 (after aging), further confirming the stability for the lattice expansion sample is better than the unexpanded counterpart. Briefly, the improvement in stability of photoexcited devices can be attributed to the reduction of defect states.

As for thermal stability (Figure 4e), the device with photoexcitation remains 67% of its initial PCE at 85 °C for 500 h under ambient air conditions of RH 25%, which is better than the 55% of the pristine counterpart. The slight improvement in thermal stability can be attributed to the lattice expansion induced by the elongation of Pb─I bond length, which promotes relaxation of local lattice strain.^[^ ^9^ ^]^ This relaxation reduces the defect density and passivates trap states in the film, thereby suppressing ion migration to some extent. As displayed in Figure 4f, we measure the stability of these unpackaged devices under 150 min of continuous light illumination under AM1.5G. The PCE of the perovskite devices without photoexcitation decreases to 75% under 150 min of illumination, while the treated devices still remain 95%. Meanwhile, after 150 min of illumination, the *V*_OC_ is increased as compared to the initial value, due to further expansion of the lattice under light. These results clearly indicate that the decomposition of the (PDA)(FA)_n‐1_Pb_n_I_3n+1_(*n* = 4) perovskite under high temperature or light illumination is inhibited to some extent in the expanded lattice.

## 3 Conclusion

We unveil the structure dynamics of 2D perovskite under photoexcitation, demonstrating the elongation of the metal─halogen bond length and reduction of the Pb 6s‐I 5p antibonding state, resulting in an expanded lattice structure and a larger bandgap. The process also enhances crystallinity by promoting Ostwald ripening and reduces both surface and bulk defects due to strong carrier‐lattice coupling. As a result, carrier lifetime is extended and the recombination rate is suppressed, ultimately contributing to a higher output. We fabricate 2D (PDA)(FA)_n‐1_Pb_n_I_3n+1_ (*n* = 4) perovskite device achieving improved performance and excellent stability through ultrafast‐photoexcitation‐induced lattice expansion. This light‐2D perovskites interaction induced surface passivation through lattice expansion not only provides a new idea for the interface passivation strategy, but also offers a new direction for the application of ultrafast laser processing.

## Conflict of Interest

The authors declare no conflict of interest.

## Supporting information



Supporting Information

## Data Availability

All data needed to present are available in the main text or the supplementary materials.
