# Supplementary material for: Ultrafast Photoexcitation Induced Nonthermal Lattice Expansion in 2D Perovskite
Source: Adv Sci (Weinh). 2025 Oct 21;13(2):e10954. doi: 10.1002/advs.202510954 (PMC12786345; doi:10.1002/advs.202510954)
Supplement: Supplementary file 1 — Supporting Information [file ADVS-13-e10954-s001.pdf]

Supporting Information

Ultrafast Photoexcitation Induced Nonthermal Lattice Expansion in 2D Perovskite

Xiangyu Chen, Jiakang Zhou, Yunfan Yue, Zhongle Zeng, Xuewen Wang
Corresponding author: xwwang@whut.edu.cn

The PDF file includes:

Materials and Methods
Supplementary Text
Figure. S1 to S23
Tables S1 to S5
References

Materials and Methods

Materials

$SnCl_2·2H_2O$ (>99.99%) and PbI_2 (>99.99%) were purchased from Aladdin and TCI, respectively. Formamidinium chloride (FACl, >99.5%), formamidinium iodide (FAI, >99.5%), 1,3-Propanediammonium iodide salt ($PDAI_2$, >98%) and 2,2′,7,7′-Tetrakis (N, N-di-p-methoxyphenylamine)-9,9′spirobifluorene (spiro-OMeTAD, >99%) were bought from Xi'an Yuri Solar Co. Ltd. HCl (37 wt%) was purchased from China National Medicines Corporation Ltd. All other chemicals including N, N-dimethylformamide (DMF, >99.8%), dimethyl sulfoxide (DMSO, >99.5%), urea (>99%), mercaptoacetic acid (>99%), 4-tertbutylpyridine (tBP, >99%), acetonitrile (ACN, >99.8%), Empirical Formula (Hill Notation) (FK209, >99%) and lithium bis (trifluoromethanesulfonyl) imide (Li-TFSI, >99%) were purchased from Sigma-Aldrich.

Methods

Preparation of perovskite film: The Fluorine doped Tin Oxide (FTO) substrates were first cleaned by dishwashing liquid to remove dirty, which were further ultrasonic cleaned sequentially with detergent solution, deionized water, and alcohol for 30 minutes. Afte drying, the substrates were cleaned with ultraviolet ozone (UVO) for 15 minutes to further remove the organic residues. Then, the substrates were immersed in the prepared diluted $SnCl_2·2H_2O$ solution with a concentration of 0.002 M for 150 minutes at 90 °C, followed by rinsing with deionized water. Subsequently, the SnO_2/FTO substrates were transferred inside the glovebox, spin-coated by 1.0 M $(PDA)(FA)_{n-1}Pb_nI_{3n+1}$ (n = 2, 4) perovskite precursor solution at 5000 rpm for 25 seconds. Chlorobenzene was added to substrates at the last 15 seconds as an antisolvent. The films were then annealed at 150 °C for 10 minutes. After cooling down, the perovskite thin films were taken out of the glovebox to further processed under different laser polishing conditions.

Ultrafast photoexcitation induced lattice expansion: The ultrafast photoexcitation process was performed on the prepared perovskite film under ambient conditions. The ultrafast laser source (Pharos-10 W, light conversion) with a center wavelength of 1030 nm and pulse duration around 260 fs was guided into a galvo scanning system (ES112166, Aerotech Inc., USA) equipped with a field lens with a focal length of 100 mm. To tune the laser pulse energy fluence, an attenuator (2-EWP-R-0515-M, Altechna) was placed in the optical path. We fix the laser fluence at 20.37 mJ/cm^2 to scan the perovskite films.

To fabricate the whole devices, the untreated and the photoexcitation treated perovskite films were then taken into the glovebox and spin-coated by Spiro-OMeTAD solution at 4000 rpm for 20 seconds. Finally, a 90 nm gold film was evaporated on the top of hole transport layer as the back electrode.

Device Characterization

The surface crystallinity of perovskite thin films was investigated using a Bruker D8 Discover X-ray diffractometer with a Co Kα radiation source (λ = 1.5406 Å). Different types of perovskite thin films were tested by a UV-Vis spectrometer (lambda 750S, PerkinElmer) to attain the optical bandgap. Photoluminescence and Time-resolved Photoluminescence spectra were carried out by Spectrofluorometer (FLS 1000, Edinburgh instruments) using 375 nm laser excitation. The TG-DSC curves were measured by the integrated thermal analyzer (Netzsch STA-2500) at a temperature rise rate of 5 °C min^{-1} in an N_2 atmosphere. The surface roughness was observed by atomic force microscopy (AFM, Nanoscope IV). The morphology of perovskite films was measured using a scanning electron microscopy (JSM-IT800). The transient absorption spectra

were conducted by HARPIA-TA, along with 400 nm pump wavelength. The chemical characterization of the thin films was analyzed by an X-ray photoelectron spectrometer (ESCALAB 250Xi). J-V measurements of the perovskite solar cells were performed using a solar simulator (Oriel 94023A, 300 W) and a Keithley 2400 source meter in the ambient atmosphere under AM 1.5G sun light (100 mW/cm^2). The intensity (100 mW/cm^2) was calibrated using a standard Si solar cell (Oriel, VLSI standards).

Supplementary Text

Perovskite threshold analysis

We systematically varied the single-pulse energy of a femtosecond laser and performed single-point exposure on perovskite films at a 1 MHz repetition rate and a wavelength of 1030 nm to compare the photodecomposition thresholds across different perovskite structures. As shown in **Figure S1**, under laser irradiation, a circular crater initially forms on the surface of perovskite film. With increasing laser fluence, stress concentration occurs in the central region, leading to the formation of cracks. This marks the end of the photodegradation stage and the onset of the ablation process. As the laser fluence further increases, these cracks ultimately develop into a hole. Both RP and 3D perovskites exhibit similar trends. We then extract the photodecomposition thresholds of three perovskite structures by analyzing the decomposition region and performing a linear fit to the fluence-dependent photodecomposition response as shown in **Figure S2**. The photodecomposition thresholds of DJ-phase, RP-phase and 3D perovskite film are 46.99 mJ cm^{-2}, 57.40 mJ cm^{-2} and 39.25 mJ cm^{-2}, respectively. The relatively low photodecomposition threshold of the 3D phase makes it prone to rapid decomposition under moderately high laser fluence, thereby precluding the observation of transient structural dynamics. Conversely, the high photodecomposition threshold of the RP phase necessitates the application of extremely high fluence to initiate structural changes, which introduces practical challenges in experimental control and increases the risk of unwanted effects. Hence, the photodecomposition resistance threshold of the DJ-phase perovskite is between those of the RP and 3D phases, making it a suitable candidate for investigating structural dynamics under high-intensity illumination.

Computational simulation details

Section 1: rt-TDDFT methods for photoexcitation

Real-time time dependent density theory (rt-TDDFT) is developed on the basis of Runge-Gross theorem,[1] time-dependent Kohn-Sham equations, adiabatic local density approximation (ALDA), and other methods of density functional theory (DFT). In this rt-TDDFT simulation, the ultrafast photoexcitation process is calculated using the ab initio computing package (PWmat). [2] In the rt-TDDFT algorithm, the time-dependent wave function, $\psi_j(t)$ is expanded by the adiabatic eigenstate, $\phi_i(t)$. The evolution of the wave function is transformed into the evolution of coefficients $C_{j,i}(t)$.

$$\psi_j(t) = \sum_i C_{j,i}(t)\phi_i(t)$$

Meanwhile, the linear time-dependent Hamiltonian (LTDH) is used to represent the evolution of the Hamiltonian within a time step.

$$H(t) = H(t_1) + \frac{t - t_1}{\Delta t}[H(t_1 + \Delta t) - H(t_1)]$$

To simulate light excitation, we add an A-field in the k-space of the Hamiltonian.

$$H(t) = \frac{1}{2}[-i\nabla + A(t)]^2$$

Due to the electric field polarization along the x, y, and z directions, the resulting Hamiltonian is,

$$H(t) = \frac{1}{2}(-i\nabla_x + A_x \times E(t))^2 + \frac{1}{2}(-i\nabla_y + A_y \times E(t))^2 + \frac{1}{2}(-i\nabla_z + A_z \times E(t))^2$$

Section 2: Laser field parameter settings

The TDDFT calculations are based on the Perdew-Burke-Ernzerhof (PBE) exchange-correlation functional and the SG15 norm-conserving pseudopotentials (NCPP). [3] By simply specifying the organic spacer cation at the A' site, the A-site ion, the B-site metal, the X-site halogen, B-X bond length and the layer number n, the two-dimensional DJ perovskite crystal structure can be automatically generated using the Pyrovskite: A software proposed by Stanton, R. and Trivedi, D. J..[4] The unit cell parameters of $(PDA)(FA)_{n-1}Pb_nI_{3n+1}$ (n = 1 and n = 4) are shown in **Table S1.** The perovskite (n = 4) structure model contains 116 atoms and perovskite (n = 1) contains 44, whose initial geometry and corresponding charge density are shown in **Figure S3**, which indicates that the charge density of iodine atoms is greater than that of lead atoms in the octahedron. At the beginning, the structure is relaxed before the TDDFT calculation to obtain a relatively stable structure. The maximum standard for atomic force convergence is set at 0.01 eV/Å. Laser pulses in the shape of Gaussian are simulated by E(t).

$$E(t) = E_0\, cos(\,\omega t)\, exp[\,-(t - t_0)^2/(2\sigma^2)]$$

E_0, electric field intensity, is a constant defined in V/Å. We can study excited electron concentrations of materials by controlling the electric field intensity of the laser via adjusting the parameters of the laser pulse as shown in **Figure S4**. In our simulation, we simulated the laser pulse with a photon energy of 2.532 eV or 1.266 eV to explore the suitable experiment parameters. For computational convenience, we set the pulse width to $\sqrt{2}\sigma = 25$ fs.

Due to the algorithmic characteristics of the software, it allows for much larger time steps (0.1 fs - 0.2 fs) compared to traditional real-time TDDFT (sub-attosecond). Therefore, a time step of 0.1 fs was set in the simulation. The wave function is expanded on a plane wave basis set with a cutoff energy of 50 Ry. The NVE ensemble was used in the simulation, characterized by constant number of atoms (N), volume (V), and total energy (E).

Section 3: Time dependent character analysis

We have simulated the condition with excited electron concentration of 1.44%. **Figure S5** shows that during excitation, the concentration of excited electrons gradually increases. Besides, the charge of the indicated lead and iodine atoms is consistently distributed in the antibonding orbitals as displayed in **Figure S5(B)**, generating Coulomb interaction to stretch bonds. The excited electrons transfer from I atoms to Pb atoms changing the charge distribution of the atoms in octahedron, displayed in **Figure S6,** weakening the covalence Pb-I bonds and prolong the bond length.

Figure S8 shows the partial density of states (PDOS) and density of states (DOS) along with the changes in Pb-I bond length and lattice temperature at various excited electron density under non-resonant 2.532 eV photon energy excitation, clearly illustrating the distribution of excited electrons and holes. It is noted that in **Figure S8(A)**, localized energy bands appear in the bandgap under excessive electron density, indicating the presence of localized carriers. The spatial localization distribution of excess photoexcited carriers concentrated at the band edge aggravate dynamic instability of the lattice, which in turn leading to the density of localized excited carriers

surpasses the needed density of phonon softening, leading to lattice temperature more than melting point and eventually causing the self-amplified melting of the system as displayed in **Figure S8(B)** (*28*). It demonstrates excessive excited carrier density damage the materials at non-resonant excitation. With the excited electron density decreasing, the Pb-I bond length prolongates and rapidly reach stable in 600 fs while the lattice temperature is below the melting point shown in **Figure S8(C)-(D)**, resulting a non-thermal expansion effect induced by Coulomb force. Intriguingly, **Figure S8(E)-(F)** reveals the system maintains a temperature below the melting point but the structure is in an unstable state with increasing bond length at lower excited density.

We also study near-resonant excitation, 1.266 eV photon energy, to explore the different corresponding changes compared to non-resonant excitation displayed in **Figure S9**. Despite the electron density is high, the bandgap remains existing and the lattice temperature is around the melting point (**Figure S9(A)-(B)**) different from non-resonant excitation. The moderate laser intensity is displayed in **Figure 1(B)** and **Figure S5(C)**, resulting a lattice temperature far below the melt point and consequently the non-thermal expansion effect. Similarly, in **Figure S9(C)-(D),** the system is in an unbalanced state with an increasing bond length even though the lattice temperature is low when laser with lower power excites less electrons. Hence, we utilize near-resonant excitation to carry on experiments.

Specifically, the blue shaded regions in **Figure S11** illustrate the distributions and energy gap between the antibonding σ^* and bonding π orbitals within the valence band maximum (VBM) under different excitation energies. By comparing the variations in these blue regions between **Figure S11 (B) and (C)** relative to **Figure S11 (A)**, the energy level schematic depicted in **Figure 1 (C)** can be verified. Furthermore, **Figure S11 (B) and (C)** reveal that the energy gap between the bonding and antibonding orbitals remains unchanged with different photon energy.

Section 4: Calculation of energy bands after expansion

To obtain more accurate bandgap in the simulation, the Heyd-Scuseria-Ernzerhof (HSE06) hybrid functional is used for the band structure calculations of the expanded lattice. The screening parameter for the HSE06 hybrid functional is OMEGA = 0.2, the mixing parameter for the short-range Fock exchange part is ALPHA = 0.15, and the mixing parameter for the long-range Fock exchange part is BETA = 0.0. After photoexcitation, the downward shift of valence band maximum (VBM) and the holding of conduction band minimum (CBM) together lead to an increase in the bandgap shown in **Figure S12**, serving as the evidence of lattice expansion.

The bandgap calculated by using HSE06 functional is closer to the experiment value. It can be seen in **Figure S17** only calculated by HSE06 functional that with the increase of expansion rate, the bandgap shows an upward trend.

X-ray photo-electron spectroscopy analysis

Pb 4f spectra of the before and after photoexcitation treated perovskite films, where two peaks near at 137.9 eV and 142.8 eV corresponding to $4f_{7/2}$ and $4f_{5/2}$ orbitals of Pb^{2+} are observed, respectively. After the photoexcitation process, the binding energy of Pb 4f spectra for the perovskite film shifted slightly towards high energy. Besides, the I 3d, C 1s and N 1s spectra have an identical tendency, indicating the change of charge density of the atoms and an expansion effect of the perovskite lattice, which is caused by the ultrafast photostriction.

TG-DSC analysis

We conducted TG-DSC analysis to measure the melting point of the DJ perovskite. For $(PDA)FA_3Pb_4I_{12}$ (n = 4) films, the first peak of slight mass decline on the TG curve is attributed to the volatilization of the halogens, and the second peak of rapid mass decline is attributed to the melting process, corresponding to the endothermic peak on the DSC curve. Thus, we can conclude the melting point of the $(PDA)FA_3Pb_4I_{12}$ (n = 4) is 610 K.

Perovskite threshold

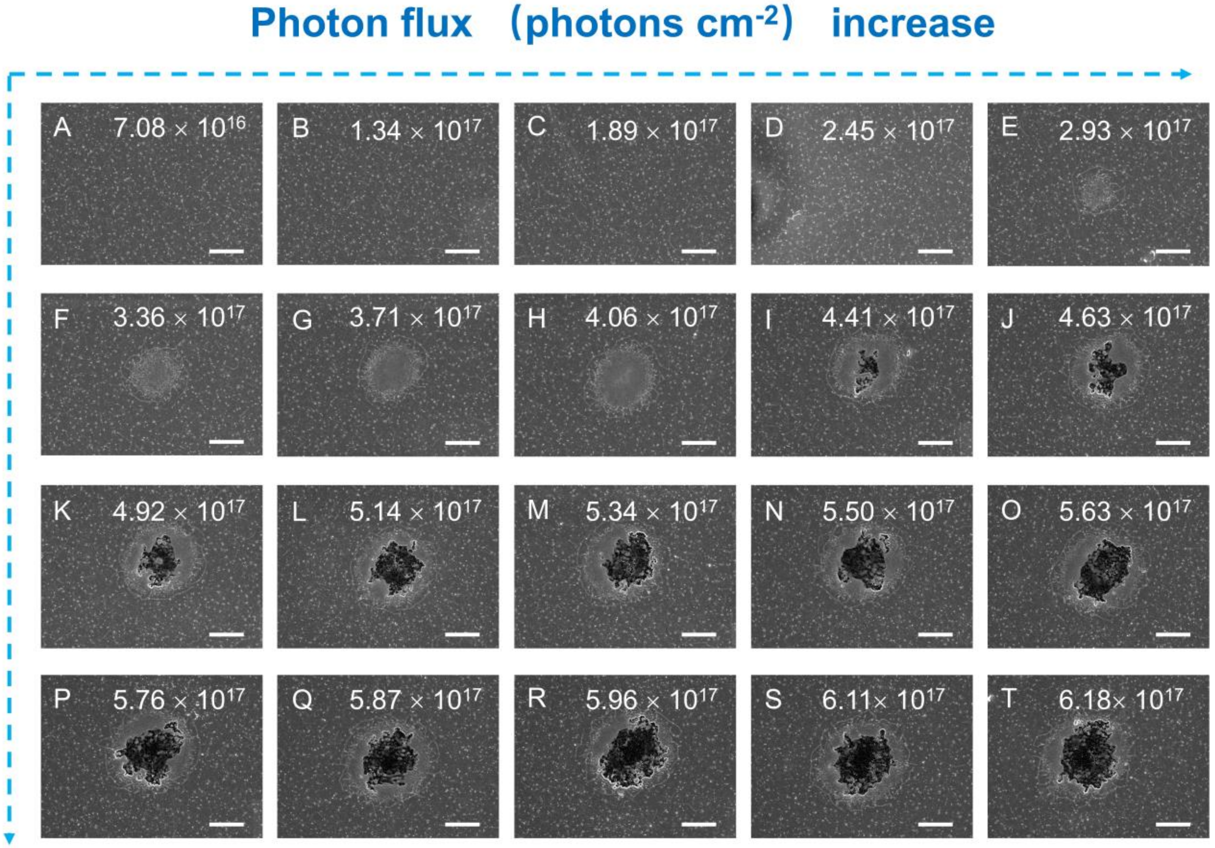

Figure S1. Morphology evolution of perovskite films under various laser fluence treatment with 1030 nm at 1 MHz repetition, scale bar: 10 μm.

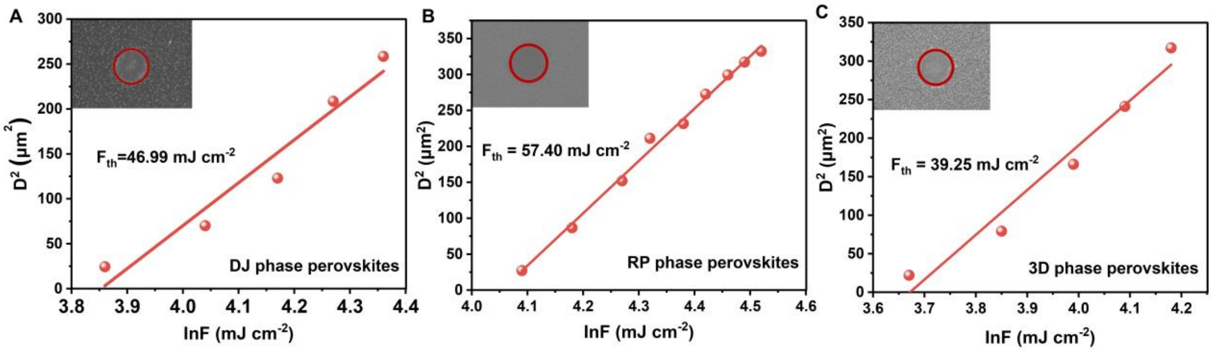

Figure S2. The square of the outer diameter D^2 as a function of the logarithm of the applied laser ablation fluence In*F*. (A) The photon decomposition threshold of the DJ $(PDA)(FA)_3Pb_4I_{13}$ film is

46.99 mJ cm^{-2}. (B) The photon decomposition threshold of the RP $(PEA)_2(MA)_3Pb_4I_{13}$ film is 57.40 mJ cm^{-2}. (C) The photon decomposition threshold of 3D $(FAPbI_3)_{0.95}(MAPbI_3)_{0.05}$ film is 39.25 mJ cm^{-2}.

Calculation results

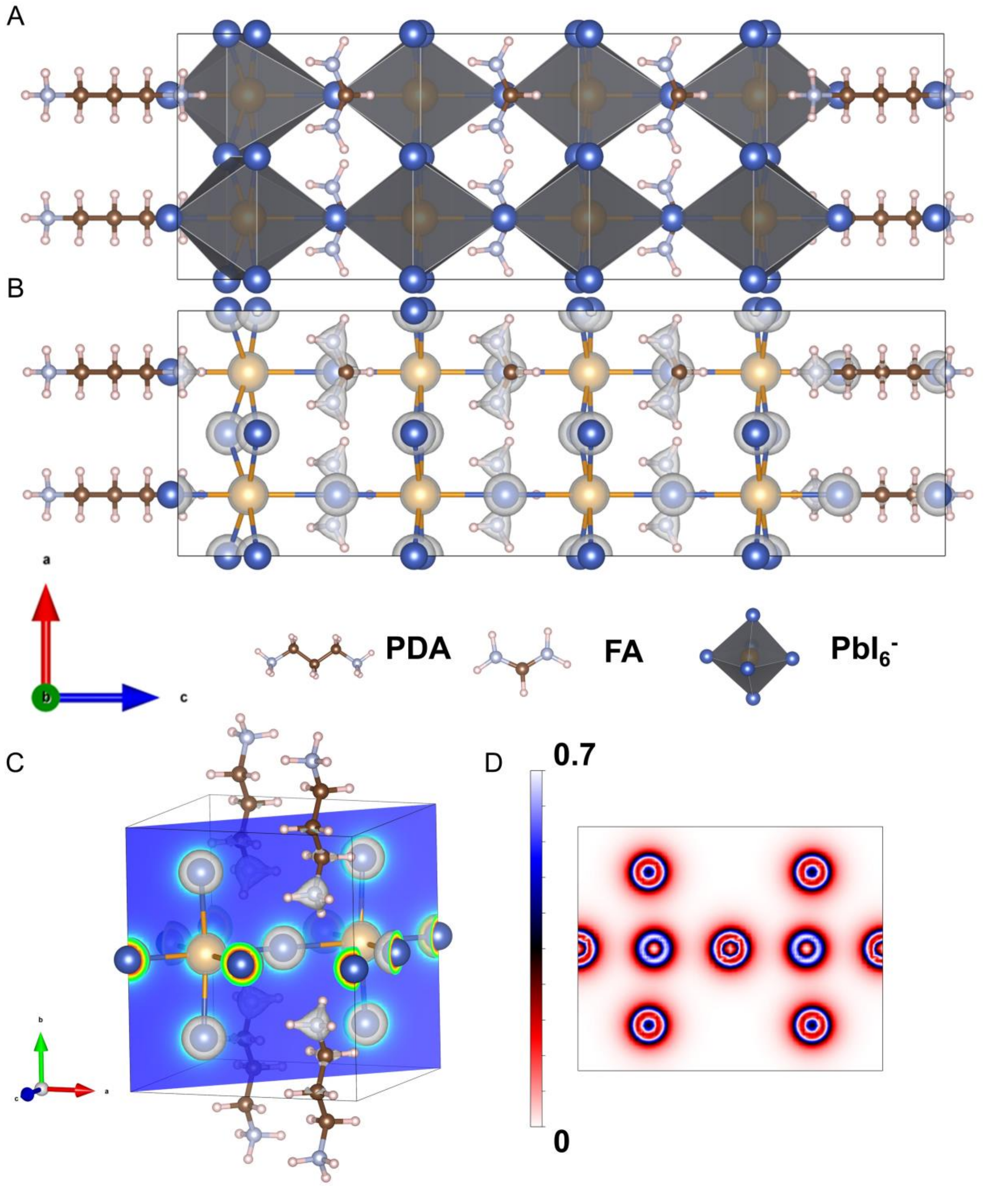

Figure S3. (**A**) Schematic diagram of the initial structure of $(PDA)(FA)_{n-1}Pb_nI_{3n+1}$ (n = 4) DJ quasi-2D perovskite. The unit cell parameters for $(PDA)(FA)_{n-1}Pb_nI_{3n+1}$ (n = 4) are shown in Table S1. (**B**) Charge density of the initial structure of n = 4 quasi-2D perovskite. The gray area represents

the charge density of the atom. The volume of the gray sphere surrounding the iodine atom is larger than that surrounding the lead atom in the octahedron, indicating the charge density around iodine atoms is higher than that around lead atoms. (**C**) Charge density of the unit cell of $PDAPbI_4$ 2D perovskite. (**D**) Charge density distribution of the (101) crystal plane of $PDAPbI_4$ 2D perovskite.

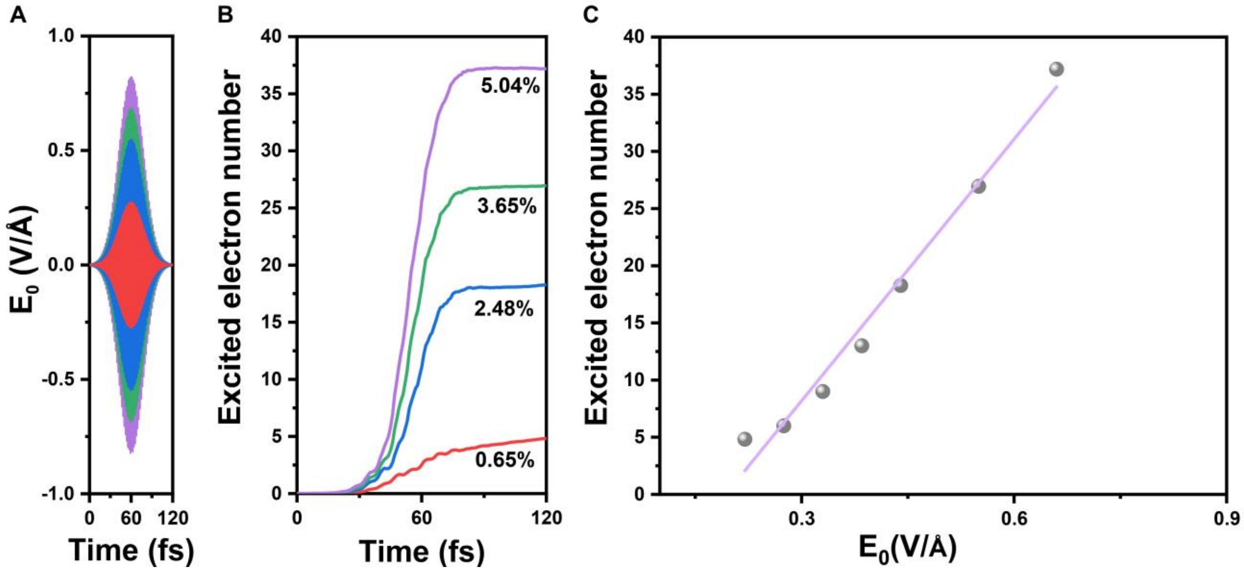

Figure S4. (**A**) Evolution of electronic field intensity E_0 induced by laser pulses with time in 120 fs. (**B**) The number of excited electrons under different E_0 as a function of time. The percentages represent the corresponding concentration of excited electrons originating from valence band. (**C**) Linear correspondence between the number of excited electrons and E_0.

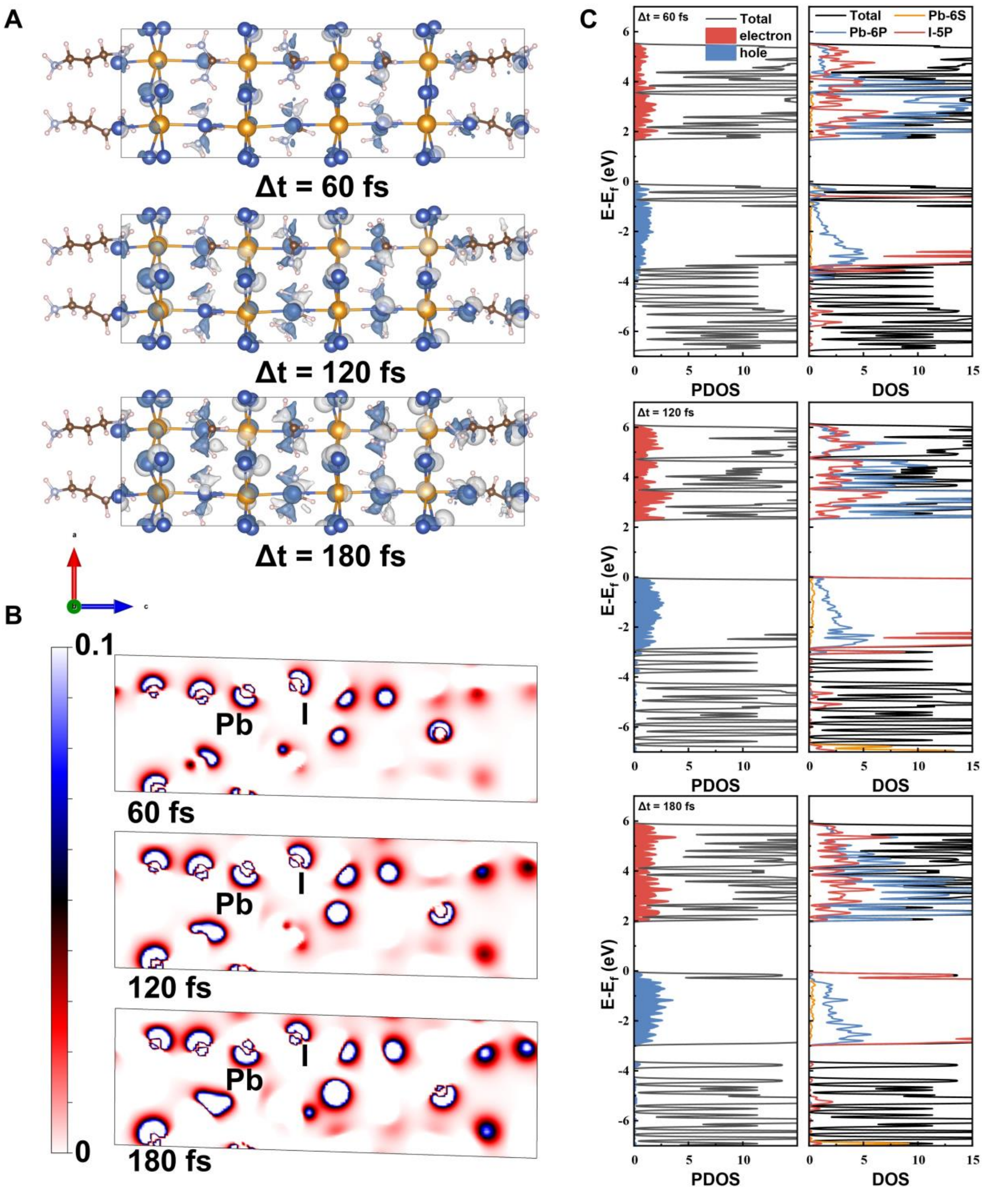

Figure S5. (**A**) Differential charge density for systems at 60 fs, 120 fs, and 180 fs with an excitation electron density of 1.44%, while 60 fs, 120 fs, and 180 fs correspond to the peak time of laser pulse intensity, the end of the laser pulse, and a period after the end of the laser pulse, respectively. (**B**) Distribution of excited electrons on the lead-iodine bond at 60 fs, 120 fs, and 180 fs. (**C**) PDOS and DOS at time of 60 fs, 120 fs, and 180 fs.

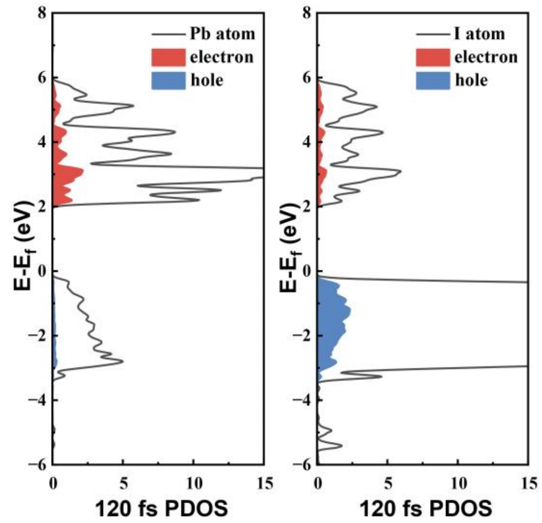

Figure S6. PDOS of atom I and Pb at 120 fs under 1.44% electron density photoexcitation with 1.266 eV photon energy, where the red reign and the blue reign represent the photoexcited electrons and holes, correspondingly.

X-ray photo-electron spectroscopy analysis

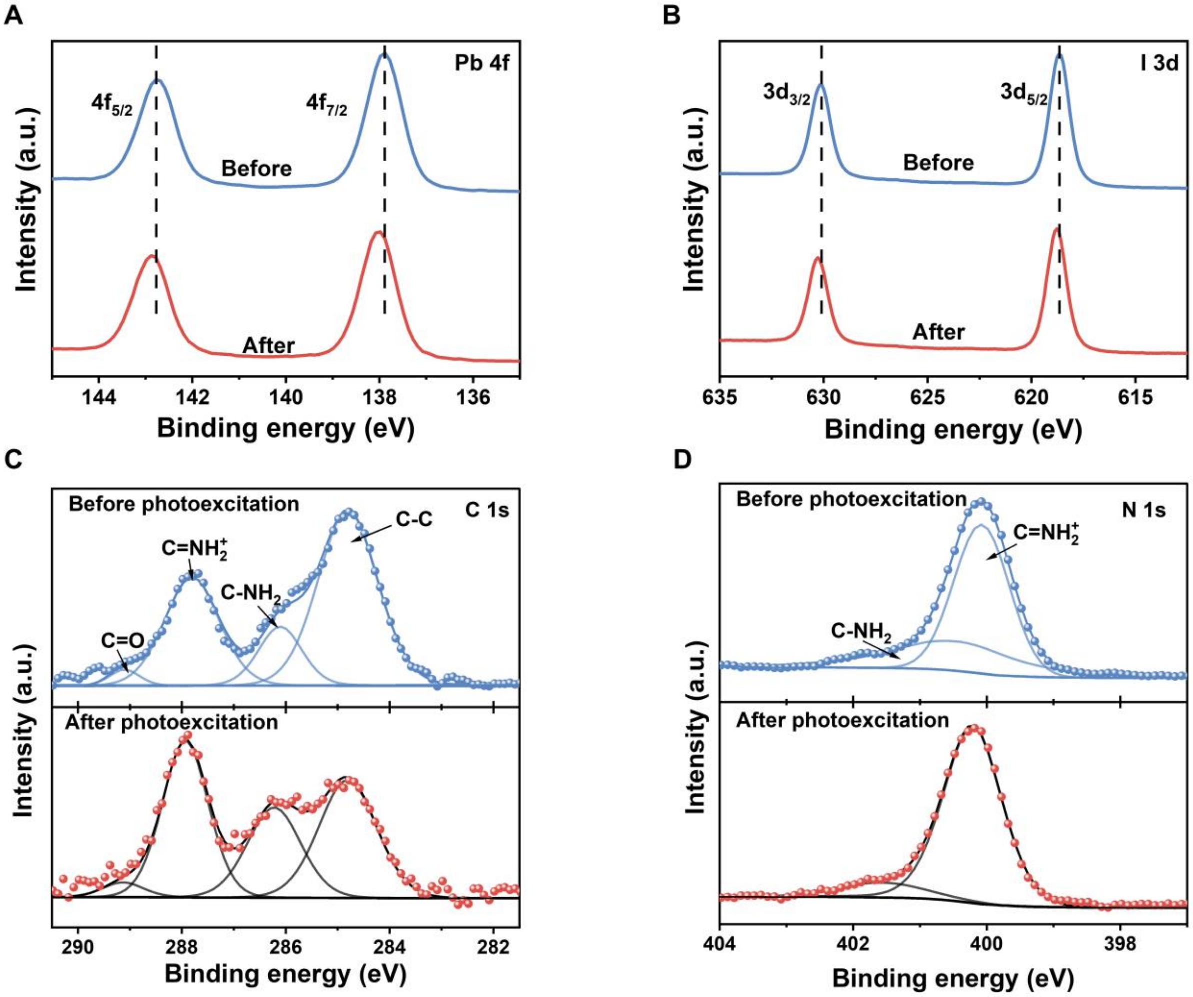

Figure S7. XPS spectra of (**A**) Pb 4f, (**B**) I 3d, (**C**) C 1s and (**D**) N 1s for the perovskite film before and after the photoexcitation.

Structural change under different excited electron density

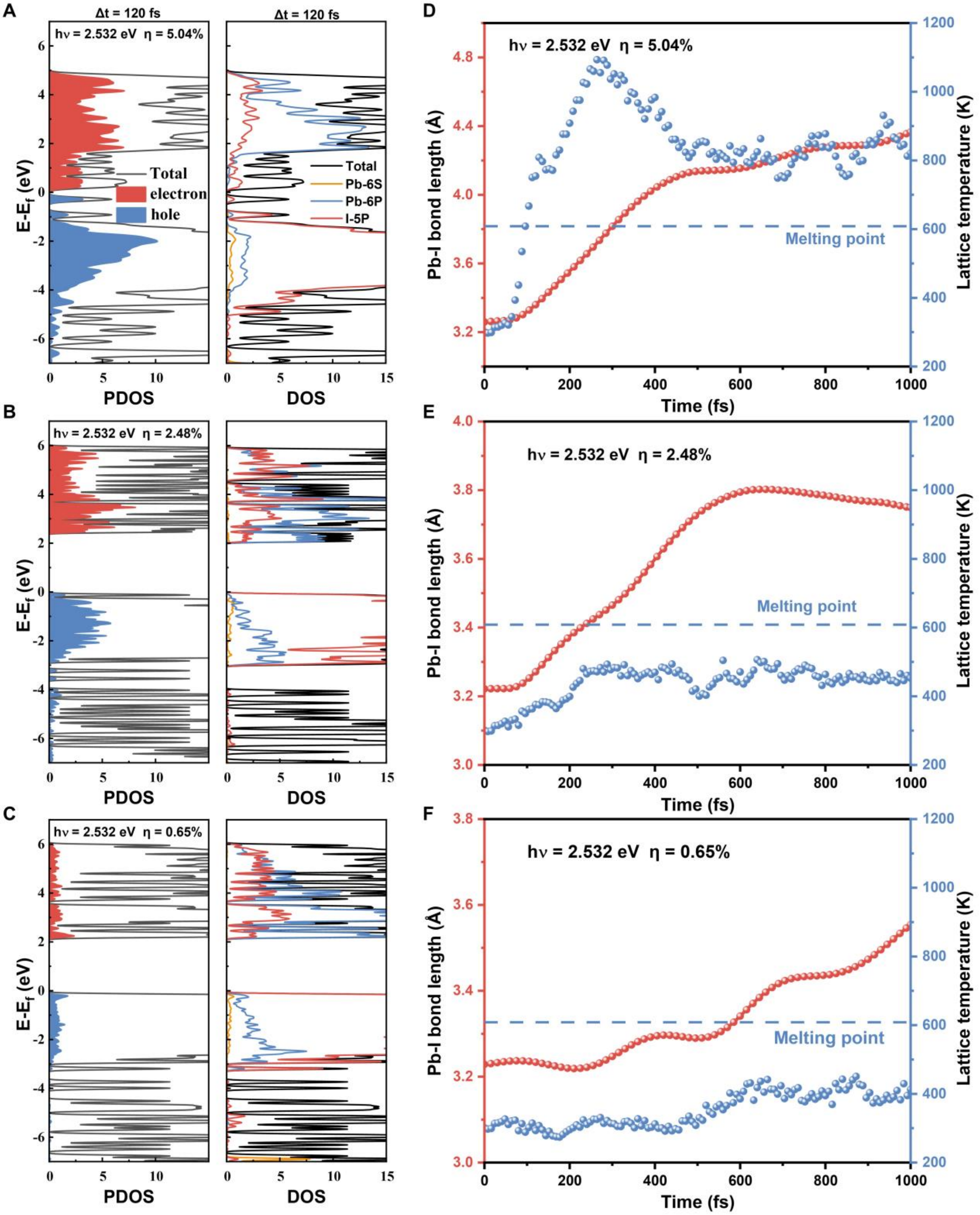

Figure S8. Under 2.532 eV non-resonant excitation photon energy, the PDOS and DOS distribution for excited electron concentration η (**A**) 5.04% (**B**) 2.48% (**C**) 0.65%. Evolution of the average lead-iodine bond length and lattice temperature of the system with time during the excitation process at a photon energy of 2.532 eV, with excited electron concentration η of (**D**) 5.04% (**E**) 2.48% (**F**) 0.65%, respectively.

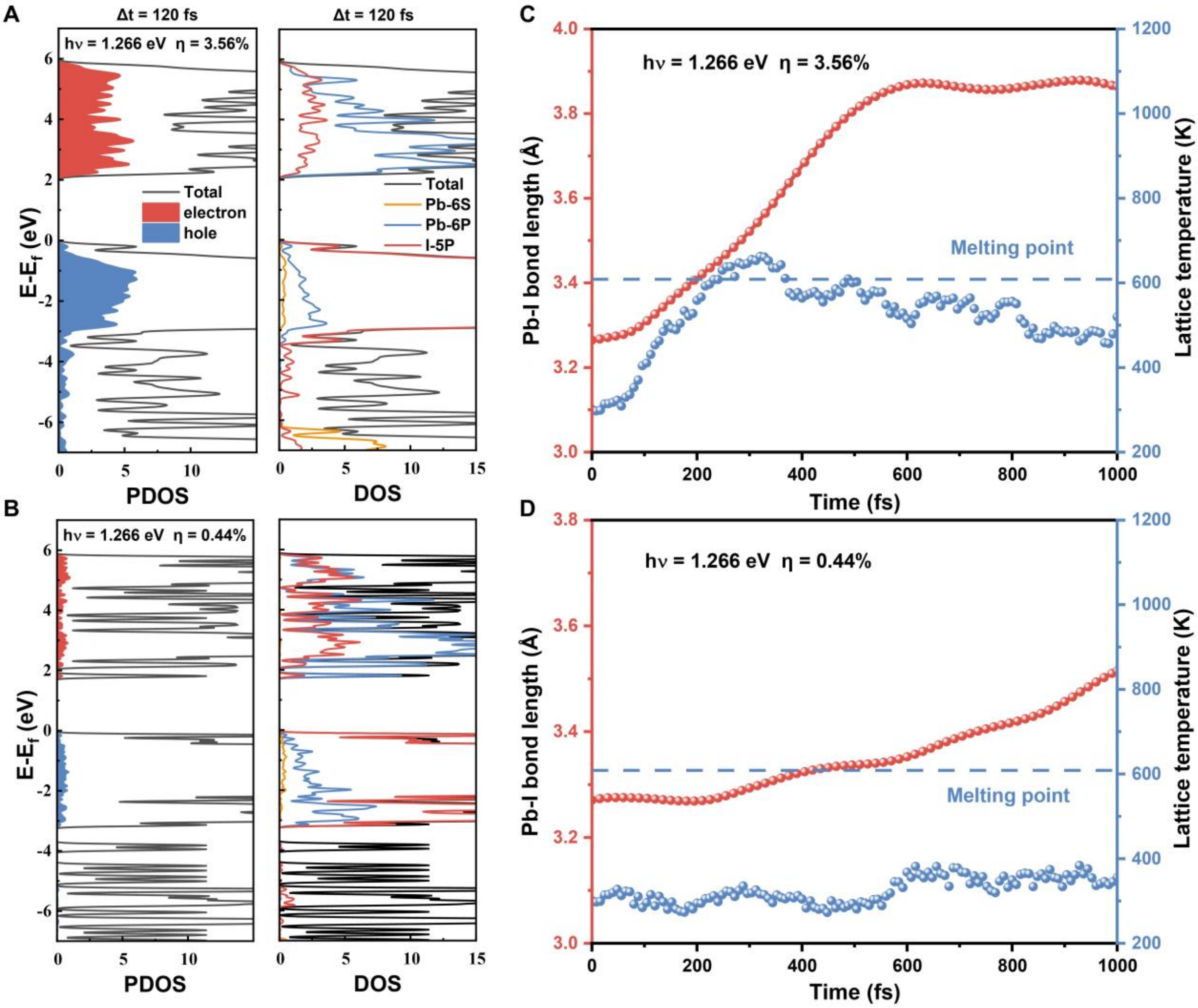

Figure S9. The PDOS and DOS distribution for excited electron concentration η (**A**) 3.56% (**B**) 0.44%, with 1.266 eV near- resonant excitation photon energy. Besides, the average Pb-I bond length and the lattice temperature as a function of time with excited electron concentration of (**C**) 3.56% (**D**) 0.44%, respectively.

TG-DSC analysis

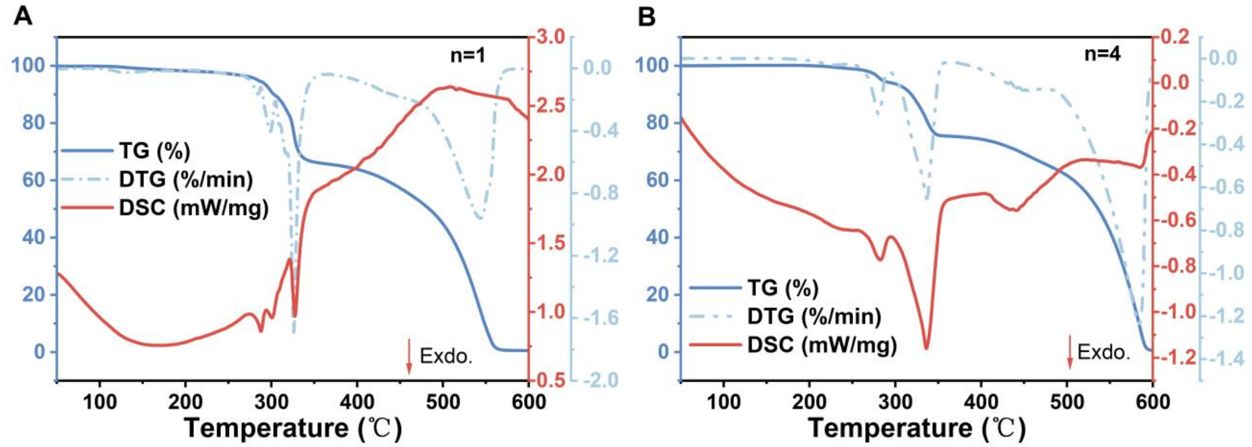

Figure S10. TG-DSC curves of the (**A**) $PDAPbI_4$ (n = 1) films and (**B**) $(PDA)FA_3Pb_4I_{12}$ (n = 4) films.

Energy gap in VBM at different excitation conditions

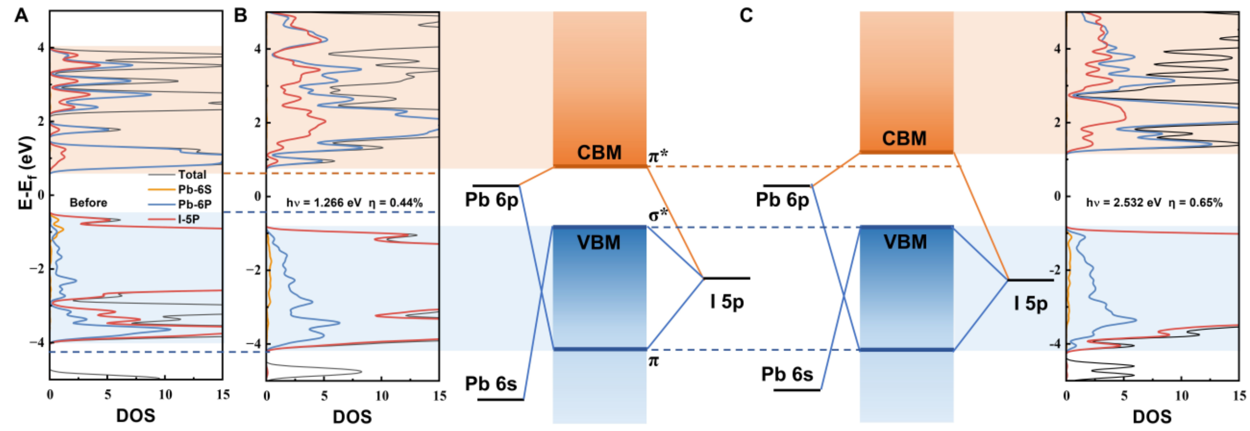

Figure S11. (**A**) DOS of pristine before photoexcitation. (**B**) Under 1.266 eV excitation photon energy, the partial density of states and the corresponding energy level schematic. (**C**) The corresponding energy level schematic and the partial density of states with 2.532 eV excitation photon energy.

Band structure

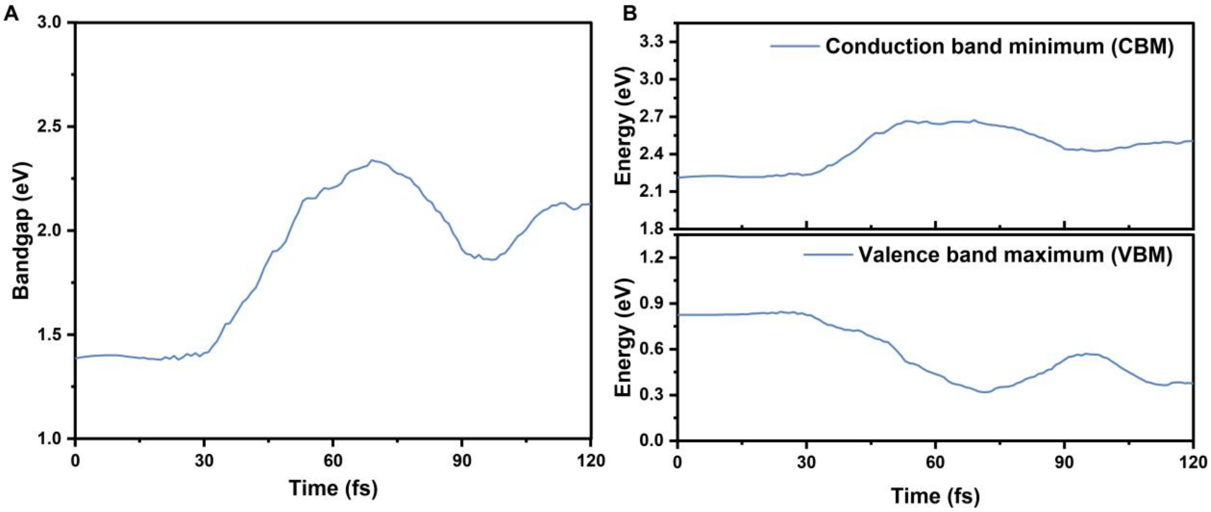

Figure S12. (**A**) The variation of the bandgap with simulation time. (**B**) Evolution of the valence band maximum and conduction band minimum versus time.

Transient absorption spectra analysis

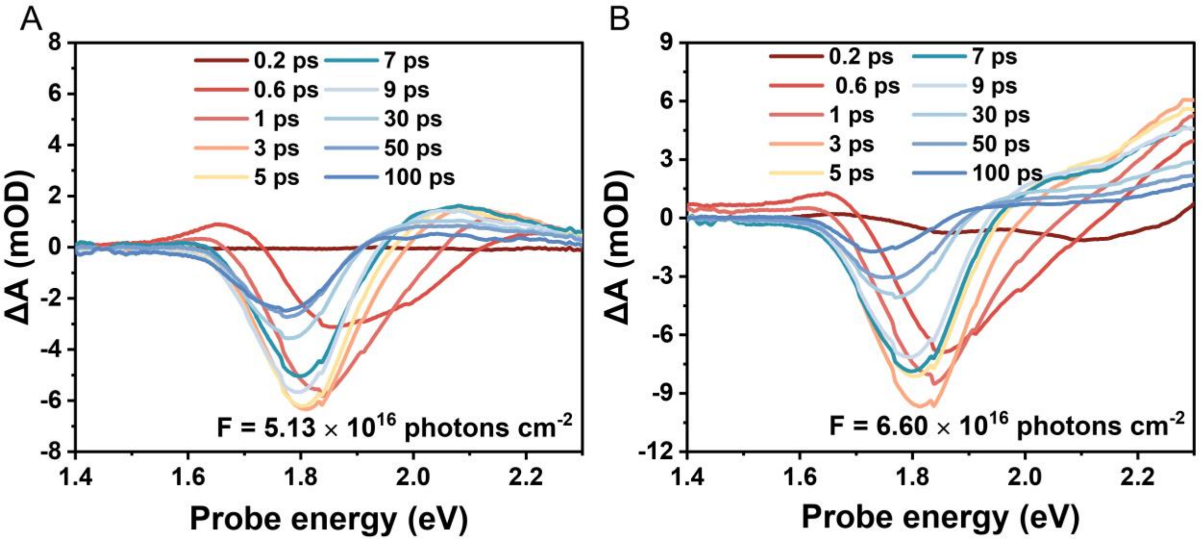

Figure S13. Carrier dynamics of perovskite film at indicated various delay time varying from 0.2 ps to 100 ps of photon flux F = 5.13 $\times 10^{16}$ photons cm^{-2} (25.46 mJ cm^{-2}) (**A**) and F = 6.60 $\times 10^{16}$ photons cm^{-2} (32.72 mJ/cm^2) (**B**).

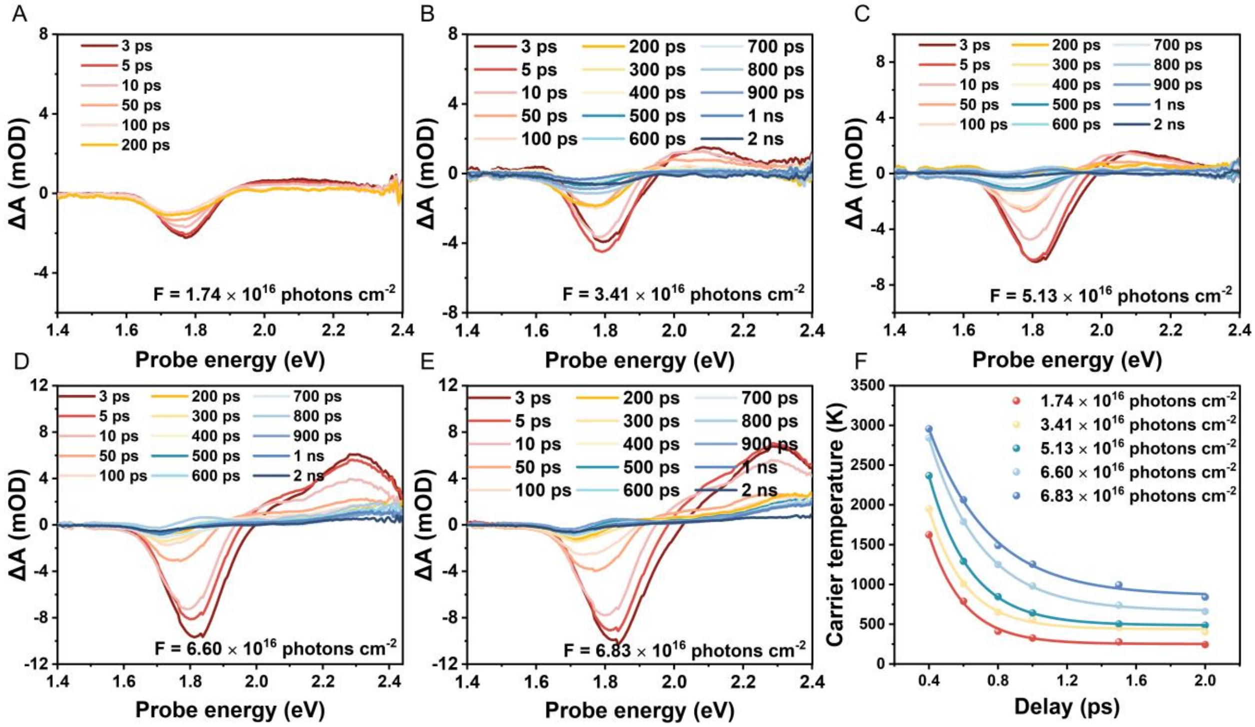

Figure S14. Carrier dynamics of perovskite film at indicated various delay time of photon flux F = 1.74 $\times 10^{16}$ photons cm^{-2} (8.65 mJ/cm^2) (**A**), F = 3.41 $\times 10^{16}$ photons cm^{-2} (16.93 mJ cm^{-2}) (**B**), F = 5.13 $\times 10^{16}$ photons cm^{-2} (25.46 mJ cm^{-2}) (**C**)and F = 6.60 $\times 10^{16}$ photons cm^{-2} (32.72 mJ/cm^2) (**D**) and F = 6.83 $\times 10^{16}$ photons cm^{-2} (33.87 mJ/cm^2) (**E**).

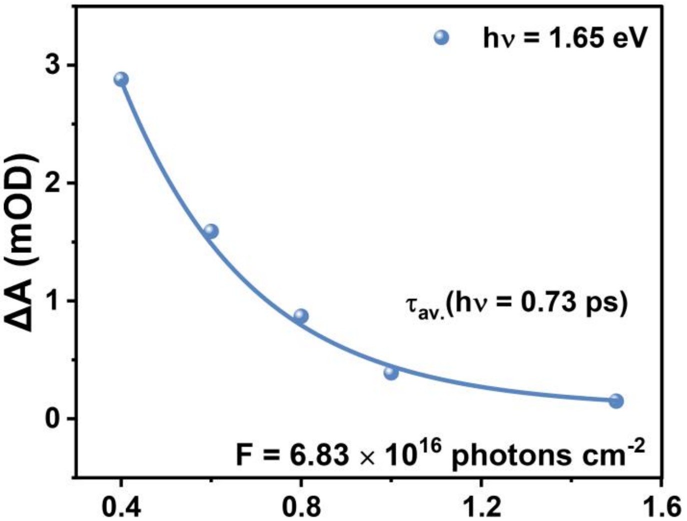

Figure S15. Graph of ΔA as a function of delay time at hν =1.65 eV of photon flux F = 6.83 × 10^{16} photons cm^{-2}.

Lattice expansion for $(PDA)FA_3Pb_4I_{12}$ (n = 4) films

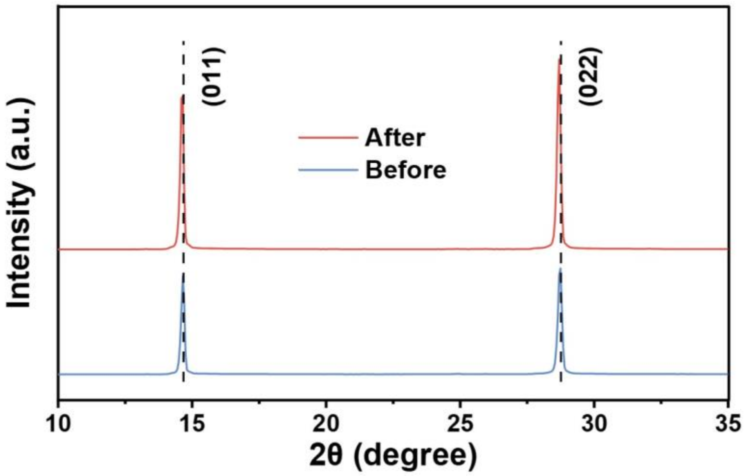

Figure S16. XRD patterns of $(PDA)FA_3Pb_4I_{12}$ (n = 4) film before and after the photoexcitation.

Bandgap at different volume expansion rates

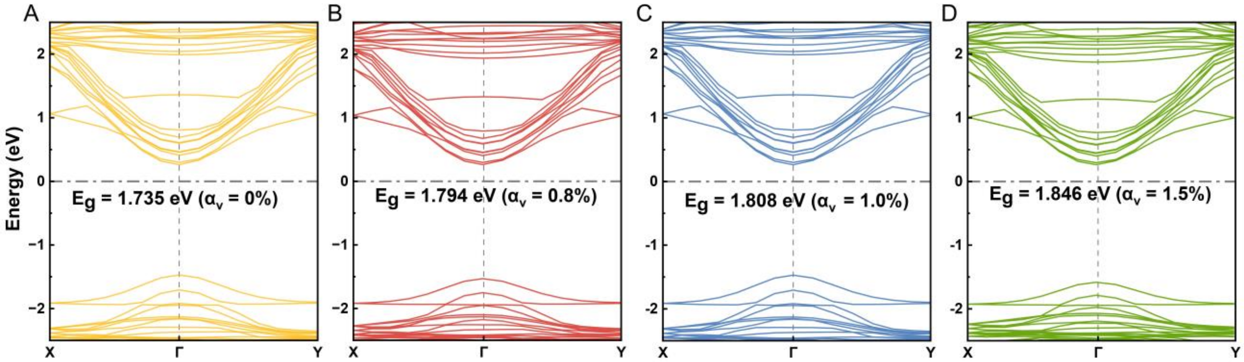

Figure S17. Simulation results of band structure by HSE06 functional for different volume expansion rates (α_v) (**A**) α_v = 0%, (**B**) α_v = 0.8%, (**C**) α_v = 1.0%, and (**D**) α_v = 1.5%.

Thin film characterization

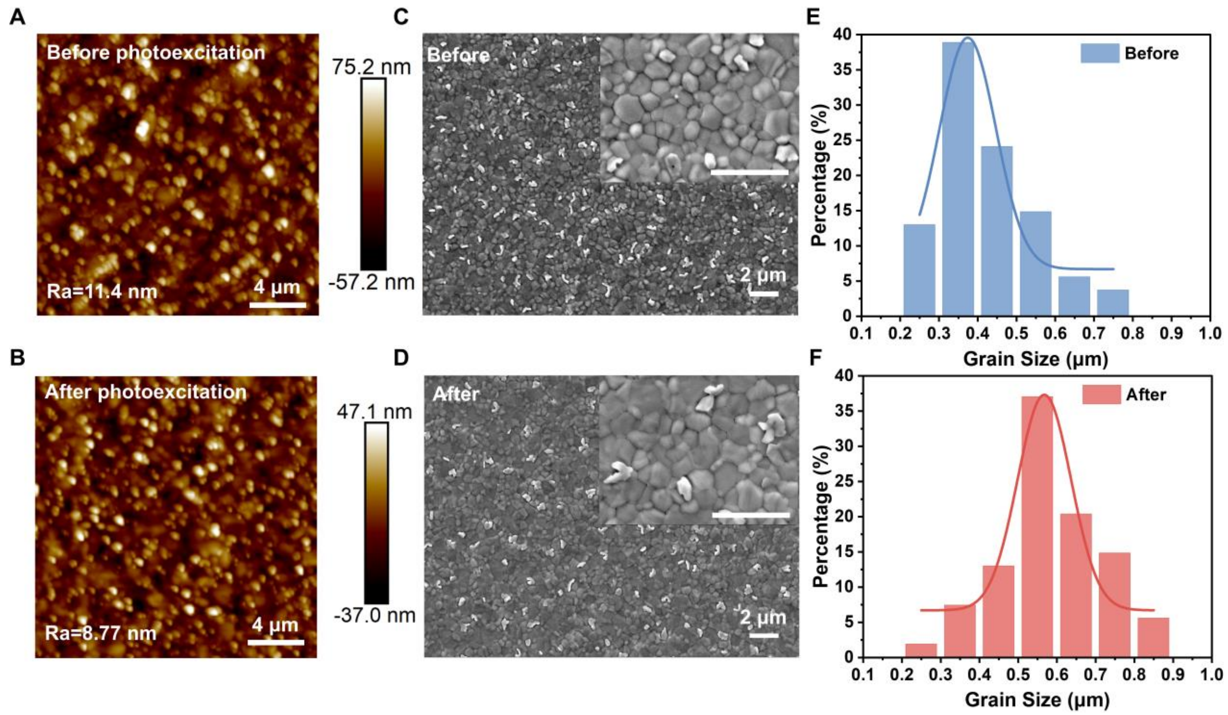

Figure S18. AFM images of the (**A**) without and the (**B**) with photoexcitation treated perovskite films. Top-view SEM surface images of the (**C**) without and the (**D**) with photoexcitation treated perovskite films. The distribution of grain size of the (**E**) without and the (**F**) with photoexcitation treated perovskite films.

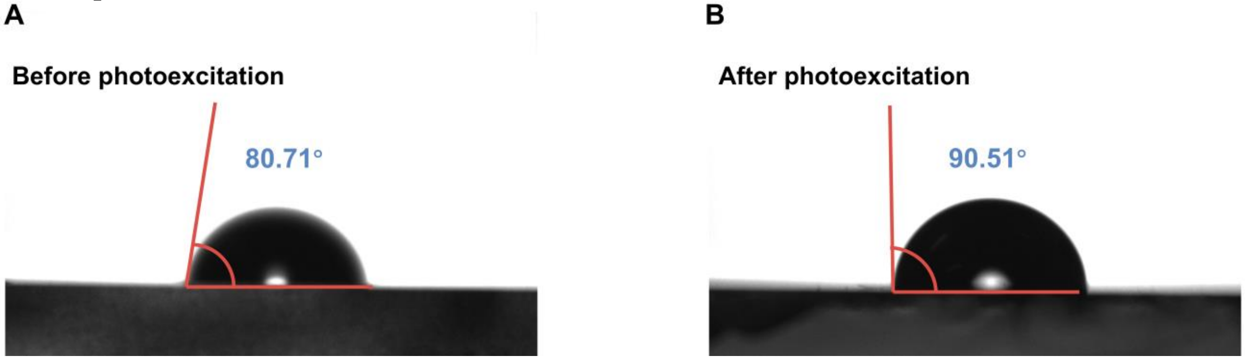

Figure S19. Contact angle of the (**A**) pristine perovskite film, (**B**) photoexcitation treated perovskite film.

Device performances

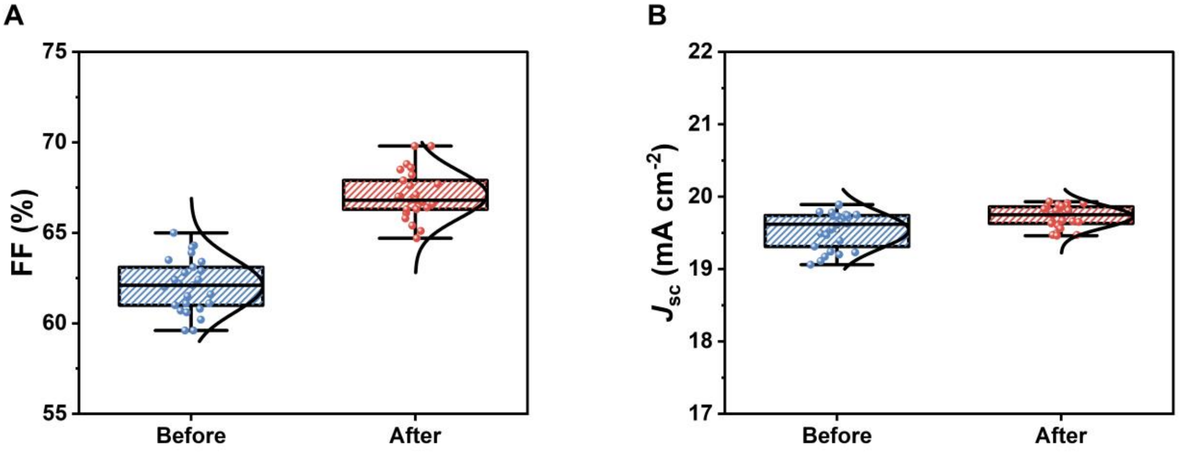

Figure S20. Statistical photovoltaic parameters of (**A**) FF, (**B**) J_{SC} and for $(PDA)(FA)_{n-1}Pb_nI_{3n+1}$(n = 2) without and with the lattice expansion.

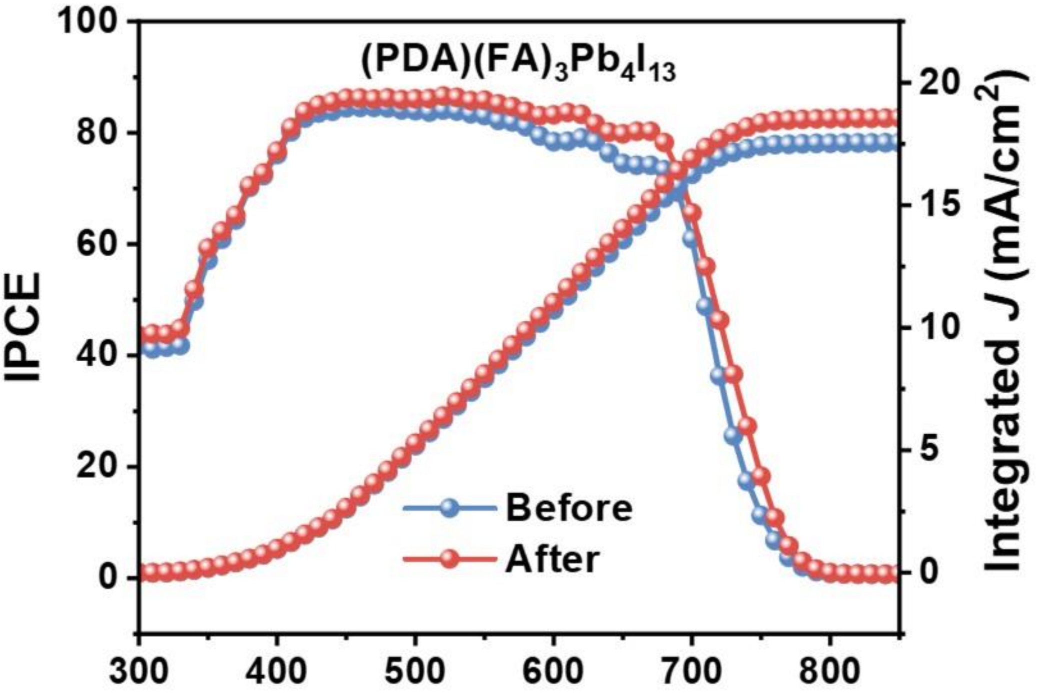

Fig. S21. The external quantum efficiency spectra of perovskite devices before and after the photoexcitation.

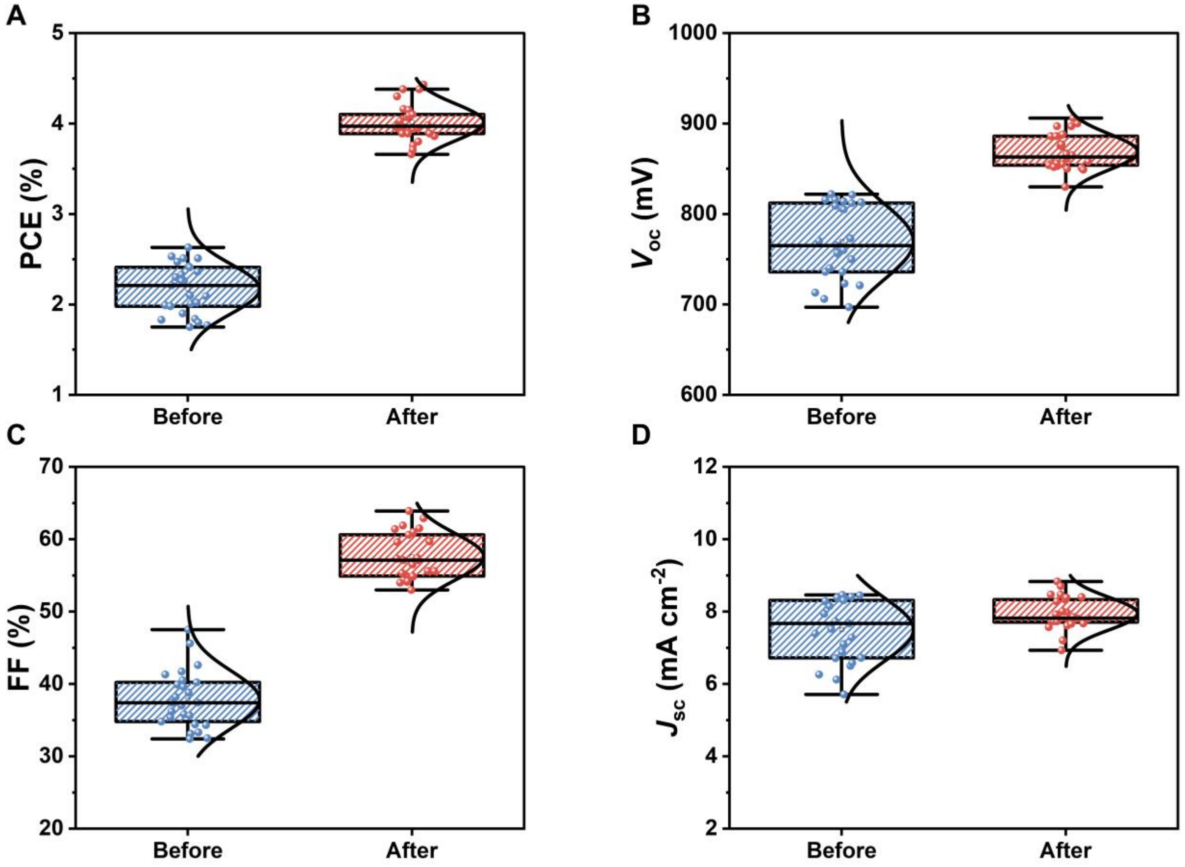

Figure S22. Statistical photovoltaic parameters of (**A**) V_{OC}, (**B**) FF, (**C**) J_{SC} and (**D**) PCE for $(PDA)(FA)_{n-1}Pb_nI_{3n+1}$(n = 2) without and with the lattice expansion.

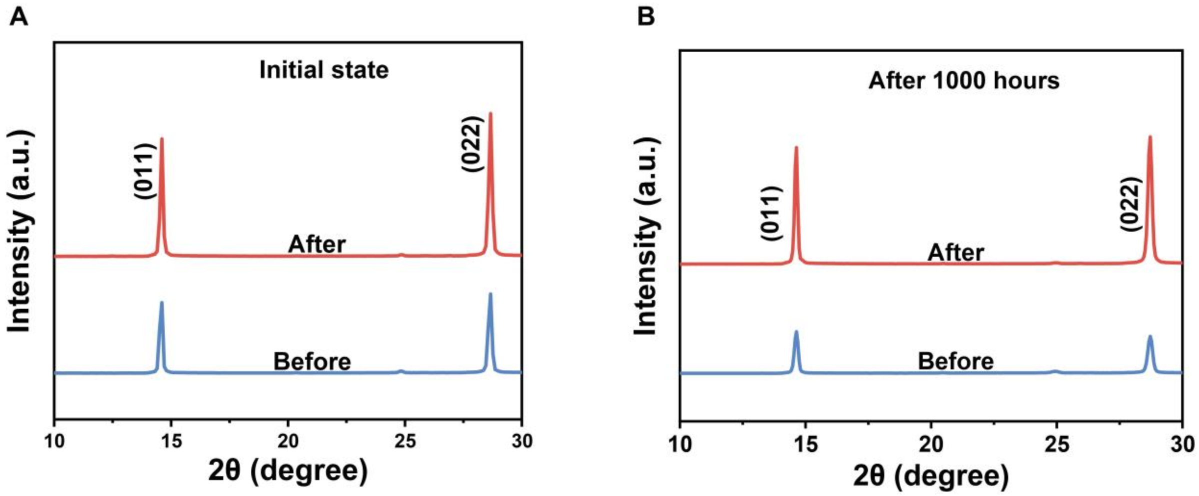

Figure S23. XRD patterns of $(PDA)(FA)_{n-1}Pb_nI_{3n+1}$ (n = 4) without and with the photoexcitation (**A**) before and (**D**) after 1000 hours.

Table S1. The unit cell parameters of $(PDA)(FA)_{n-1}Pb_nI_{3n+1}$ ((n = 1 and n = 4).

	n = 1	n = 4
Crystal system	orthorhombic	orthorhombic
a (Å)	9.07	9.26
b (Å)	9.97	8.65
c (Å)	8.52	28.92
α	90°	90°
β	90°	90°
γ	90°	90°

Table S2. Carrier density corresponding to different photon flux. The carrier density is estimated through $n = \frac{F\alpha}{h\nu}$, where F is the pump fluence, α is the absorption coefficient of the film, and hν is the photon energy. [5]

Photon flux (photons cm^{-2})	Carrier density (cm^{-3})
1.74×10^{16}	4.01×10^{21}
3.41×10^{16}	7.84×10^{21}
5.13×10^{16}	1.18×10^{22}
6.60×10^{16}	1.52×10^{22}
6.83×10^{16}	1.57×10^{22}

Table S3. The measured formation and decay of the PIA features located at 2.1 eV and 2.3 eV are fitted by the following equation: $\Delta A(t) = -A_1\exp(-t/\tau_1) + A_2\exp(-t/\tau_2) + A_3\exp(-t/\tau_3)$, where A_1, A_2 and A_3 are the relative amplitudes, τ_1 is the formation time constant, and τ_2 and τ_3 are the decay time constant. Average carrier lifetime (τ_{av}) can be calculated by the equation of $\tau_{av} = (A_1\tau_1^2 + A_2\tau_2^2 + A_3\tau_3^2)/(A_1\tau_1 + A_2\tau_2 + A_3\tau_3)$, thus the τ_{av} at 2.1 eV and 2.3 eV is 882.32 ps and 1741.15 ps, respectively.

PIA	A_1	A_2	A_3	τ_1 (ps)	τ_2 (ps)	τ_3 (ps)
2.1 eV	8.29	3.92	1.01	2.10	20.00	967.21
2.3 eV	8.58	5.50	0.49	0.67	17.23	1924.96

Table S4. The measured formation and decay of the GSB signal are fitted by the following equation: $\Delta A(t) = -A_1\exp(-t/\tau_1) + A_2\exp(-t/\tau_2) + A_3\exp(-t/\tau_3)$, where A_1, A_2 and A_3 are the relative amplitudes, τ_1 is the formation time constant, and τ_2 and τ_3 are the decay time constant. Average carrier lifetime (τ_{av}) can be calculated by the equation of $\tau_{av} = (A_1\tau_1^2 + A_2\tau_2^2 + A_3\tau_3^2)/(A_1\tau_1 + A_2\tau_2 + A_3\tau_3)$, thus the τ_{av} before and after the lattice expansion is 60.43 ps and 80.13 ps, respectively.

Sample	A_1	A_2	A_3	τ_1 (ps)	τ_2 (ps)	τ_3 (ps)
Before	-2.94	0.64	0.53	0.46	8.61	66.27
After	-3.23	0.63	0.49	0.41	13.18	90.38

Table S5. Bi-exponential decay equation of $I(t) = I_0 + A_1\exp(-t/\tau_1) + A_2\exp(-t/\tau_2)$ was used to fit the TRPL data, where I_0 is a constant, A_1 and A_2 are the relative amplitudes, while τ_1 and τ_2 are the time constants related to monomolecular non-radiative recombination and the bimolecular radiative recombination, respectively. Besides, average carrier lifetime (τ_{av}) can be estimated by the equation of $\tau_{av} = (A_1\tau_1^2 + A_2\tau_2^2)/(A_1\tau_1 + A_2\tau_2)$.

Sample	A_1	A_2	τ_1 (ns)	τ_2 (ns)	τ_{av} (ns)
Glass/Perovskite-After	0.56	0.42	4.67	19.25	15.67
Glass/Perovskite-Before	0.65	0.33	3.86	16.67	12.69

Table S6. The measured champion photovoltaic parameters under one sun illumination (100 mW cm^{-2}) for the PSCs based on the $(PDA)(FA)_{n-1}Pb_nI_{3n+1}$ (n = 2) perovskite films without and with the photoexcitation.

Treatment	V_{OC} (V)	J_{SC} (mA cm^{-2})	FF (%)	PCE (%)
Before	806	6.88	47.5	2.63
After	900	7.81	62.9	4.43

References

1 E. Runge, E.K.U. Gross, Density-functional theory for time-dependent systems. *Phys. Rev. Lett*. **52**, 997 (1984).

2 W. J. A. B, Z. C. A, et al., The analysis of a plane wave pseudopotential density functional theory code on a GPU machine. Comput. *Phys. Commun*. **184**, 9-18 (2013).

3 D. R. Hamann, Optimized norm-conserving Vanderbilt pseudopotentials. *Phys. Rev. B* **88**, 085117 (2013).

4. R. Stanton, D. J. Trivedi, Pyrovskite: A software package for the high-throughput construction, analysis, and featurization of two- and three-dimensional perovskite systems. J. Chem. Phys. 159, 064803 (2023).

5. Yin, J., Naphade, R., Maity, P. et al. Manipulation of hot carrier cooling dynamics in two-dimensional Dion–Jacobson hybrid perovskites via Rashba band splitting. Nat Commun 12, 3995 (2021).
